# Photoexcitation of perovskite precursor solution to induce high-valent iodoplumbate species for wide bandgap perovskite solar cells with enhanced photocurrent

**DOI:** 10.1038/s41598-023-32468-w

**Published:** 2023-04-14

**Authors:** Atittaya Naikaew, Taweewat Krajangsang, Ladda Srathongsian, Chaowaphat Seriwattanachai, Patawee Sakata, Supavudh Burimart, Kanyanee Sanglee, Kittikhun Khotmungkhun, Pipat Ruankham, Suwat Romphosri, Amornrat Limmanee, Pongsakorn Kanjanaboos

**Affiliations:** 1 National Energy Technology Center (ENTEC), National Science and Technology Development Agency, Pathum Thani, 12120 Thailand; 2 School of Materials Science and Innovation, Faculty of Science, Mahidol University, Nakhon Pathom, 73170 Thailand; 3 Department of Physics and Materials Science, Faculty of Science, Chiang Mai University, Chiang Mai, 50200 Thailand; 4 Center of Excellence for Innovation in Chemistry (PERCH-CIC), Ministry of Higher Education, Science, Research and Innovation, Bangkok, 10400 Thailand

**Keywords:** Solar cells, Electronic materials

## Abstract

Solution-processed organic–inorganic hybrid perovskite solar cells are among the candidates to replace the traditional silicon solar cells due to their excellent power conversion efficiency (PCE). Despite this considerable progress, understanding the properties of the perovskite precursor solution is critical for perovskite solar cells (PSCs) to achieve high performance and reproducibility. However, the exploration of perovskite precursor chemistry and its effects on photovoltaic performances has been limited thus far. Herein, we modified the equilibrium of chemical species inside the precursor solution using different photoenergy and heat pathways to identify the corresponding perovskite film formation. The illuminated perovskite precursors exhibited a higher density of high-valent iodoplumbate species, resulting in the fabricated perovskite films with reduced defect density and uniform distribution. Conclusively, the perovskite solar cells prepared by the photoaged precursor solution had not only improved PCE but also enhanced current density, confirmed by device performance, conductive atomic force microscopy (C-AFM), and external quantum efficiency (EQE). This innovative precursor photoexcitation is a simple and effective physical process for boosting perovskite morphology and current density.

## Introduction

Hybrid organic–inorganic perovskite materials are innovative materials beneficial for countless applications due to minimal materials usage along with great practical impact. Within a short period of time, organic–inorganic lead halide perovskites have reached record certified power conversion efficiencies (PCEs) now exceeding 25.7%^1^ unprecedented in the photovoltaics field. Perovskite materials have demonstrated powerful applications in solar cells and have gained tremendous attention for various applications in optoelectronics. The exceptional efficiency outputs of perovskite solar cells are due to their excellent materials properties^2^ which include high optical absorption coefficient^3^, long-balanced charge carrier diffusion length^4–7^, low exciton binding energies, simple of band gaps tuning^7,8^ via substitutions of the precursor components. Among the perovskite materials, FAPbI_3_-based perovskite exhibits high charge-carrier extraction and a broadening absorption into the near-infrared because of their band gap (1.48 eV) which is closer to the optimum value of a single-junction solar cell^9–11^. However, the stability can be an issue in these cells. FAPbI_3_-type structure commonly has two phase structures: a perovskite black α-phase and a non-perovskite yellow δ-phase. Only the α-phase perovskite is a suitable photoactive phase^9,12^ while this phase readily transforms to the yellow δ-phase because of the large size of the FA cation. Therefore, inhibition of phase transformation can be accomplished by substituting some FA cation species with MA or Cs cation^9^. As one of the discovered solution, the three-cation system (Cs, MA, and FA cation species, triple cation perovskite) provides high PCE exceeding 21% and, at the same time, high stability over 250 h under operational conditions^13^. The large size difference between Cs cation and FA cation pushes FA cation into the favorable black α-phase perovskite, simultaneously the MA cation, which is smaller than FA cation, induces the slower FAPbI_3_ crystallization rate; however, a small fraction of the yellow δ-phase can be permitted^13^. Well-defined morphology and high crystallinity are two crucial factors for high-efficiency output^14,15^, which can be achieved via the choice of perovskite precursor^16^, solvent selection^15^, fabrication method^17^, annealing conditions^18,19^, surface passivation^20^, and engineering additives^21^. However, the maximum possible PCE of a single-junction solar cell is still governed by the Shockley-Queisser (S-Q) efficiency limit^22^. Since 2014, many researchers have paid attention to multi-junction photovoltaics or tandem solar cells^23^, which absorb multiple segments of lights to perform photon/electron conversion exceeding that of the S-Q efficiency limit for a single junction solar cell^24^. Due to perovskite’s tunable band gaps and low temperature solution processability, perovskite materials are model nominees as a top cell of the tandem solar cell. As perovskite fabrication is based on precursor properties and their drying mechanics, it is essential to understand the characteristics of the perovskite precursor solution to achieve high performance and reproducibility for further applying to tandem solar technologies. In general, the lead halide perovskite precursor solution is regarded as colloid rather than true solution, which was proved by Tyndall effect using green laser light^25^. The resulting perovskite film’s quality can be determined by colloid population and interaction within the perovskite solution. Within a colloidal solution, the organic component plays a crucial role in coordination with the inorganic component to form a lead polyhalide framework. These coordination complex components primarily determine the quality of deposited thin perovskite films such as film surface morphology, grain size, and crystallinity. When dissolving lead iodide (PbI_2_) in solvent, iodide ions (I^−^) and solvent (S) coordinate around the Pb^2+^ center, leading to the formation of various iodoplumbate complexes, i.e., PbIS_5_^+^, PbI_2_S_4_, PbI_3_S_3_^−^, PbI_4_S_2_^2−^, PbI_5_S_3_^−^, and PbI_6_^4−^. Rahimnejad et al. studied the effects of solvents used to dissolve perovskite on formation of iodoplumbate colloid (PbI_6_^4−^), which lead to high PCE and can mainly be observed at high precursor concentrations^26^. As iodoplumbates and their coordination environment play a vital role in perovskite film quality, the control of high-valent iodoplumbate population is crucial; its amount can be gained under certain conditions which are adding excess I_3_^26^, heat, or light energy illumination inside perovskite precursor solution^27^. In this work, we explore different pathways to simulate various colloid environments within perovskite precursor solution and identify the optoelectronic effects stemmed from different colloid environments. The focus is on semi-transparent perovskite material with bandgap of 1.68 eV, proper for tandem solar application The resultant materials are also investigated for both outdoor and indoor photovoltaic applications under 1 sun irradiation (AM1.5G, light intensity IL: 100 mW/cm^2^) and LED (illuminance: 1000 lux, light intensity IL: 0.31 mW/cm^2^). To show the potential for tandem solar application, transparent ITO electrode was used. We achieved a PCE up to 17.9% with a cell area of 0.25 cm^2^ and 11.2% with a cell area of 1.00 cm^2^ under 1 sun illumination. Increasing the density of high-valent iodoplumbates corresponds to better perovskite formation by reducing disorder and iodide vacancy defects, therefore improving performance. Our photoexcitation method aims to be a simple tool for refining perovskite materials via the change of colloidal environment for various solar cell applications.

## Experimental section

### Perovskite film preparation

The main triple cation perovskite formula Cs_0.05_FA_0.73_MA_0.22_Pb(I_0.77_Br_0.23_)_3_ was fabricated by the procedure described in Supplementary Information and the previous work^28^; other types of perovskites such as MAPbI_3_, Cs_0.17_FA_0.83_PbI_2.49_Br_0.51_ (CsFA), and Cs_0.05_FA_0.81_MA_0.14_PbI_2.55_Br_0.45_ (CsFAMA) were also fabricated. (See materials and experimental details in Supplementary Information). The semi-transparent perovskite can be obtained by tuning the composition between I^−^ and Br^−^ ratio (confirmed by UV–Vis spectra and optical bandgap as shown in Fig. S1a, b). 1.5 M triple cation perovskite solutions were divided by four sets for each experiment. The solution was placed at room temperature (RT) for 30 min under N_2_ environment in a glovebox as a control sample. Three other sets of solutions were placed under different excitations: (1) on hotplate at 60 $^\circ$C for 30 min, (2) under UV light at 0.015 mW/cm^2^ at 25 °C for 30 min, and (3) under 1 sun illumination at 100 mW/cm^2^ at 25 °C for 30 min prior to the film deposition in N_2_ environment. The samples were abbreviated as RT, 60 $^\circ$C, UV, and 1-Sun. To study the dynamics after light excitation, excited perovskite solutions were investigated and spin casted after specific different time durations (0, 10, 30, 90, and 360 min). To study the phase stability and the evolution of I- and Br-rich regions in the wide bandgap triple cation perovskite layer, PL measurement of all perovskite films was conducted after different time durations (0, 5, 10, 30, and 60 min) under 1 sun illumination. We performed additional experiments to assess the stability of all perovskite devices under 1 sun illumination. To accelerate the degradation of the devices, we stored them in a humidity-controlled dry box at a relative humidity of 40–60% and room temperature for 45 days without encapsulation. All experimental conditions were summarized in Fig. 2a.

### Perovskite solar cell fabrication

The 2.5 cm by 2.5 cm FTO/SnO_2_ substrates were prepared from SnCl_2_·2H_2_O powder dissolved in ethanol at 0.2 M and kept for 2 days at room temperature before use. Then, the solution was deposited onto FTO glass via spin coating at 3000 rpm with initial acceleration of 1500 rpm/s for 30 s under ambient conditions and annealed at 180 $^\circ$C for 1 h and cooled down under room temperature. This method was used in our previous publication^29^. Prior to perovskite deposition, FTO/SnO_2_ substrates were treated in a UV ozone cleaner for surface cleaning. 50 µl of triple cation perovskite solution (Cs_0.05_FA_0.73_MA_0.22_Pb(I_0.77_Br_0.23_)_3_) was then spread on the substrate and spun using one-step spin coating process at 3500 rpm for 35 s with 700 rpm/s acceleration. 100 µl anisole was then dripped on the film at 30 s as the anti-solvent after starting the program. The films were then annealed at 100 °C for 30 min. The whole spinning and annealing processes were done under a N_2_-filled glovebox. Spiro-OMeTAD as hole transport material (HTM) was prepared by dissolving 80 mg of spiro-OMeTAD in 1 ml of chlorobenzene; 28.5 µl of 4-tert-butylpyridine and 17.5 µl of Li-TFSI solution (520 mg in 1 ml acetonitrile) were added into the spiro-OMeTAD solution and stirred overnight at room temperature. The solution with the volume of 60 $\mu$l was dropped and rested for 30 s on top of the perovskite layer before starting the spin-coating process with spin speed of 2000 rpm for 30 s at 1000 rpm/s acceleration. The deposited samples were kept in a glovebox for overnight. To make 80-μm-thick carbon electrode, commercial carbon ink was doctor bladed on a glass slide and soaked in ethanol for two hours; the carbon layer could then be peeled off, dried at room temperature, and cut for further usage^30,31^. Approximately 0.04 cm^2^ of square carbon sheet was placed on top of HTM and then covered by ITO glass. Eventually, the whole stack was pressed at 0.6 MPa at 60 $^\circ$C for 5 min to finish the full device.

### Characterization methods

X-ray diffraction measurements were carried out by Bruker D8 Discover X-ray diffractometer (Cu anode material, detector scan mode using a step size of 0.01°, 0.4 s per step, and 2θ from 5° to 45°). Zetasizer Helix Particle Analyzer from Malvern Panalytical was employed to detect the size distribution. Surface morphologies and cross-sections were observed by scanning electron microscopy (SEM; JSM-7610FPlus JEOL, tungsten filament electron source, 20 kV, and secondary electron mode). The optical absorption spectra were obtained by using a UV–Vis spectrophotometer (Shimadzu UV-2600, 900–300 nm, medium mode, and absorbance mode). The Photoluminescence spectra were recorded by Horiba FluoroMax4 + spectrofluorometer (integration time of 0.1 s, excitation of 500 nm, excitation slit of 10 nm, emission wavelength measurement between 650 and 850 nm, and emission slit of 5 nm). FTIR measurements were collected by Nicolet iS50, Thermo Scientific, USA in a range between 400 and 4000 cm^−1^ with resolution of 4 cm^−1^ and 64 scans. Short-circuit current and open-circuit voltage maps were performed by the conductive atomic force microscopy (C-AFM) from Park NX10 with an ANSCMPC conductive probe (coated platinum (Ptlr5), k = 0.036 N/m, and resonance frequency of 15 kHz). Conductive measurements were done under the room environment with a scan speed of 2.5 μm/s with a contact force of 1.6 nN at sample biases of 0 V for short circuit current mapping and − 0.6 V for open circuit voltage mapping under the white light irradiation with the power of 0.2 mW/cm^2^. For stability testing, films were kept in the dark and stored in a humidity control dry box. The relative humidity (RH) was fluctuated, ranging from 40% to 60%. 1 sun irradiation (100 mW/cm^2^) was provided by AAA-class 7520-LED light source with LSS-7120 LED controller (VeraSol). 4 W LED 6500 K (Philipe, E27, cool daylight) was used as an indoor light source. The light intensity was calibrated by Si diode (Hamamatsu S1133). Solar cell performances were measured by Keithley 2400 source meter under 1 sun irradiation (100 mW/cm^2^) and indoor light at 1000 lux (0.31 mW/cm^2^) with the active area of each cell of 0.04 cm^2^ for carbon electrode along with 0.25 cm^2^ and 1.00 cm^2^ for ITO electrode. The photocurrent density–voltage (J-V) curves were measured from 1.20 V to − 0.10 V under indoor light and 1 sun with a scan step of 0.01 V and a delay time of 0 s. The measurements were done under ambient air at room temperature without any encapsulation. EQE, responsivity, and specific detectivity were measured using Enlitech QE-R quantum efficiency analyzer (DC mode with 0.04 mm^2^ beam diameter). ITO transparent electrodes were fabricated by three different processes: (1) radio frequency (RF) sputtered indium oxide/tin oxide target (In_2_O_3_/SnO_2_ = 95:5 wt %) with power supply of 13.56 MHz, sputter plasma power of 250 W, and the chamber pressure of 20 mTorr, while keeping the substrate temperature at 180 °C under Ar gas flow, (2) direct current (DC) sputtered indium oxide/tin oxide target, and (3) direct current (DC) sputtered indium oxide/tin oxide target with the mixed Ar and O_2_ (O_2_/Ar = 3.4%) gas flow. The sputter times were chosen to achieve a layer thickness of ∼80 nm.

## Results and discussion

### Crystal structure and phase transformation

**Figure 1:**
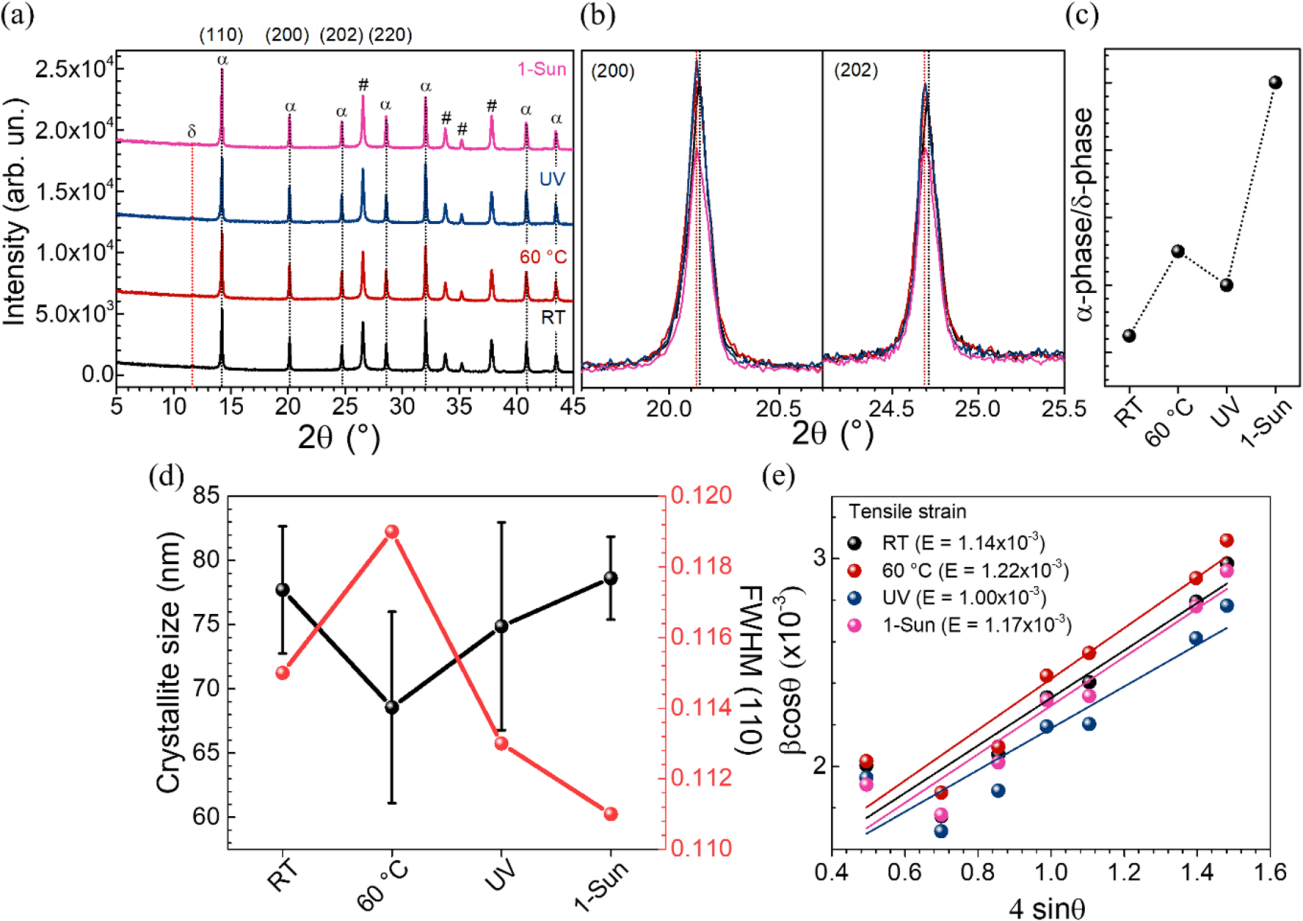
(**a**) X-ray diffraction patterns of Cs_0.05_FA_0.73_MA_0.22_Pb(I_0.77_Br_0.23_)_3_ films with different stimuli applied into perovskite solutions, where active perovskite phase (⍺), inactive perovskite phase (δ), and FTO substrate (#) signatures are labeled. (**b**) Narrow view of (200) and (202) perovskite crystal planes. (**c**) The ratio of the active phase and the inactive perovskite phase intensities measured at 14.21° and 11.57°. (**d**) Crystallite sizes and (110) full-width at half maximum (FWHM). (**e**) Williamson-Hall analysis of perovskite films with different applied stimuli.

**Figure 2:**
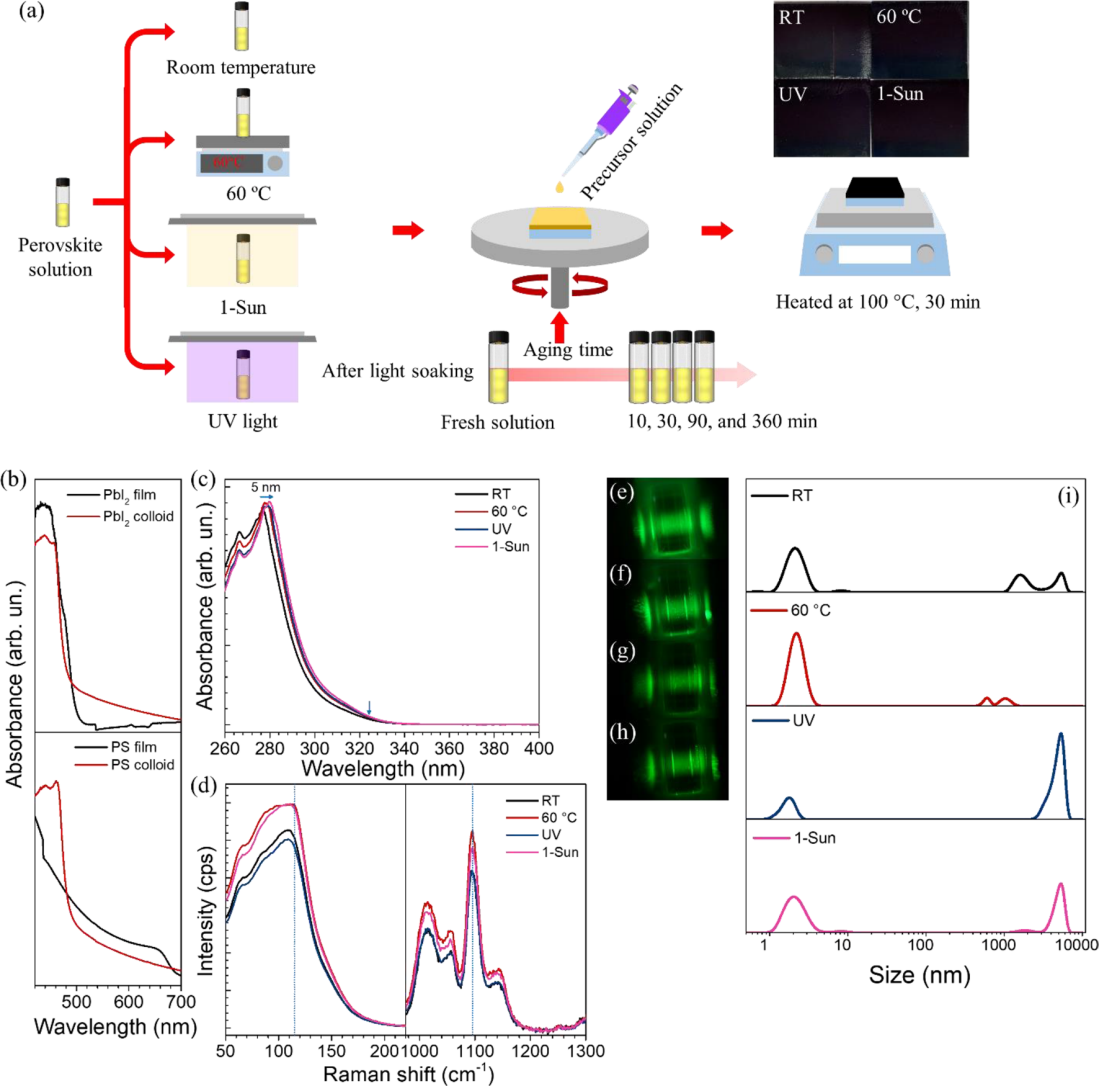
(**a**) Energy stimuli applied to perovskite solutions and film photographs. (**b**) UV–Vis spectra of thin films (black) and colloids (red) for PbI_2_ (top) and perovskite (bottom). (**c**) UV–Vis spectra of perovskite precursors with different energy stimuli applied to perovskite solutions. (**d**) Raman spectra of different perovskite thin films. Tyndall effect photographs of (**e**) RT, (**f**) 60 °C, (**g**) UV, and (**h**) 1-Sun samples. (**i**) Size distribution by dynamic light scattering.

We fabricated a series of Cs_0.05_FA_0.73_MA_0.22_Pb(I_0.77_Br_0.23_)_3_ perovskite films by varying different types of energy stimuli applied to perovskite solutions prior to the fabrication process. SEM cross-section of perovskite film is shown in Fig. S1c. Figure S2a (Supplementary Information) shows photographs of the basic precursor solutions of different energy stimuli, which are absolutely yellow and transparent to the naked eyes. The perovskite film prepared from the perovskite solution without the excitation is abbreviated as RT. The perovskite films prepared from applied different stimuli such as heat, UV, and 1 sun light illumination are called 60 °C, UV, and 1-Sun, respectively. Figure 2a shows that film photographs for all conditions have similar dark black colors. As shown in Fig. 1a, the peak positions of the black α-phase perovskite appear at 14.21$^\circ$ (110), 20.13° (200), 24.70$^\circ$ (202), 28.60$^\circ$ (220) 32.05$^\circ$ (312), 40.87$^\circ$ (224), and 43.46$^\circ$ (314)^32,33^. We also observe another peak at 11.57$^\circ$ of the non-perovskite yellow δ-phase, as this phase is more stable than the active black α-phase of FAPbI_3_ at room temperature^34^. The narrow view of perovskite crystal planes measured between 10° and 16° is shown in Fig. S2b. For the main (110) peak at 14.21$^\circ$, there is no peak shifting observed for all conditions. Interestingly, the dominant diffraction peak intensity of perovskite films prepared from the solution with UV illumination is higher than to that of RT sample by 10%, indicating higher crystallinity and reduced phase impurity^27^. The peak intensity of 60 $^\circ$C sample is similar to that of the UV sample. For 1-Sun sample, the sharp (110) diffraction peak at 14.21° is intensified by 30% as a result of more ordered crystal formation compared to that of RT. Narrow view of (200) and (202) perovskite crystal planes are shown in Fig. 1b. However, perovskite films prepared from UV and 1-Sun solutions display shifts towards lower diffraction angles at 20.12° (200) and 24.69° (202), respectively, referring to structural expansion in the unit cell parameters caused by the chemical species in a precursor solution change with illumination. We hypothesize that a peak shift originates from the transformation to the orthorhombic crystal structure with space group *Amm2*, corresponding to α-FASnI_3_ perovskite phase^35^ within the triple cation perovskite. The existence of the inactive perovskite phase (δ) can be emphasized by the ratio of active phase and inactive perovskite phase intensities measured at 14.21° and 11.57° (⍺-phase/δ-phase) as shown in Fig. 1c. For the 1-Sun sample, the ratio is much higher than those from other conditions, illustrating best film quality from the treatment. Figure 1d illustrates wider full-width at half maximum (FWHM) at (110) for 60 $^\circ$C compared to that of the RT sample; the broadening is explained by a microscale structural inhomogeneity,^36^ likely due to faster crystallization with elevated temperature. FWHMs are decreased to 0.114° and 0.110° from 0.116° for UV and 1-Sun films, respectively. Both values are smaller compared to that of the RT sample, implying improvement in crystallinity and/or more preferred (110) orientation with inactive perovskite phase suppression. The black line in Fig. 1d illustrates average crystallite size of each specific lattice plane according to the Scherrer equation. The lattice strains of perovskite films after different stimuli are estimated using the Williamson-Hall analysis. As shown in Fig. 1e, the lattice strains are 1.14 × 10^–3^ and 1.22 × 10^–3^ for RT and 60 °C, showing increased strain with treatment. However, the strain value decreases to 1.00 × 10^–3^ upon UV light treatment. The lattice strain value of 1-Sun sample is between those of 60 °C- and UV-samples. Lower lattice strain typically links to high stability and charge transport^37^. Nishimura et al*.* reported that carrier mobility is related to lattice strain, which affects carrier collection^38^. Carrier extractions can be enhanced by the decreasing strains in Pb perovskite layer^39^. UV light soaking possibly changes the chemical environment at grain boundaries, therefore reducing lattice strain. By minimizing the strain on the lattice, the formation of defect centers or traps that may capture charge carriers and have an adverse impact on the efficiency of the solar cell can be reduced^40^.

### Energy stimuli and perovskite colloidal solutions

Figure 2a is a diagram summarizing different energy stimuli and experimental flows of this work. All perovskite films have dark-brown colors, which are quite similar to the naked eye. To investigate the structural information, the optical absorptions of the colloids and their corresponding films are compared. As shown in Fig. 2b, the colloidal solutions show the plateau in absorption spectra caused by [PbI_4_]^2−^, [PbI_5_]^3−^, and [PbI_6_]^4−^ in the solution. The [PbI_6_]^4−^ compound represents the full coordination compound with a free octahedral structure, leading to the formation of ordered octahedral cluster, while the other lower coordination levels of complexes tend to share halogen ions in the form of corner-sharing to complete coordination, resulting in a soft framework of weakly interactive constituents and smaller colloidal size^25^. The absorption spectra of colloids have the blue shifted absorption edges compared to that of the corresponding film for RT condition, which explains the small colloidal size compared to the physical grain. Moreover, the perovskite colloidal solution displays much more red shift plateau absorption edge at about 490 nm than that of the pure PbI_2_ colloidal solution (470 nm). This results suggest that the perovskite solution is made of a series of new coordination compounds including those compounds between PbI_2_ and salts dissolved in solvent^25^. Figure 2c displays the absorption spectra for different colloidal precursors with the concentration of 15 mM right after different stimuli, indicating the colloidal feature with the plateau for all conditions. However, the red-shifted absorption edge by 5 nm is observed for the 1 sun illumination, suggesting larger colloidal size and therefore more [PbI_6_]^4−^. To understand the precursors chemistry of the different solutions, Raman spectroscopy was done on the original concentration solutions at 1.5 M. As shown in Fig. 2d, the precursor solutions exhibited two Raman shifts, approximately 114 and 1095 cm^-1^. All perovskite precursor solutions show a peak at 1095 cm^-1^, indicating that the perovskite precursors are dissolved in the mixed solvents. Generally, the characteristic Pb-I vibration band appears in the range 114–121 cm^-1^^41^. The peak shifts from 114 cm^-1^ for RT to 118 cm^-1^ for 1 sun light illumination with the higher intensity, indicating that the precursor solutions could absorb the imposed 1 sun light to form high-valent iodoplumbate ([PbI_6_]^4−^) inside the perovskite solution, as the peak related to [PbI_6_]^4−^ complexes appears at 118 cm^-1^^27^. The typical Tyndall effect, as depicted in Fig. 2e–h, refers to the scattering of light by particles in a colloidal solution, confirming colloidal dispersions in all perovskite precursor solutions. To determine the actual colloidal sizes, a Zeta sizer was used. Generally, the dispersed-phase compounds have well-defined diameters between approximately 2 nm and 5000 nm (see Fig. 2i). Specifically, the colloidal sizes are changed with different stimuli and resultant changes in kinetic energies. RT and 60 °C heated solutions achieve large proportions near 2 nm, in contrast, the UV and 1 sun treated-solutions display large proportions near both 2 nm and 5000 nm, indicating the formation of 1D rods with a diameter of 2 nm and a length of 5000 nm. These results further confirm that UV and 1 sun light stimuli can introduce [PbI_6_]^4−^ in the form of large colloidal rods. UV is highly effective in creating [PbI_6_]^4−^ rods, considering the UV power of 0.015 mW/cm^2^ compared to 100 mW/cm^2^ of 1 sun. With much higher power, 1 sun can generate most [PbI_6_]^4−^ population in our experiments. We hypothesize that low-valent iodoplumbate complexes are continuously converted into high-valent iodoplumbate complexes when perovskite precursor solution was illuminated by 1 sun. Presumably, DMF solvent contributes to uncontrolled nucleation and crystal growth^42^. Light energies detach DMF solvent from Pb (substitution of DMF vacancy with I^-^ to generate the high-iodoplumbate^27^), leading to high-quality perovskite films with reduced internal disorder and less iodide vacancy defects. The mechanism is in good agreement with the previous reports^27,43,44^, which demonstrate that high-valent iodoplumbate species cause high-quality perovskite films by decreasing donor defects such as iodide vacancies.

### Optical properties

**Figure 3:**
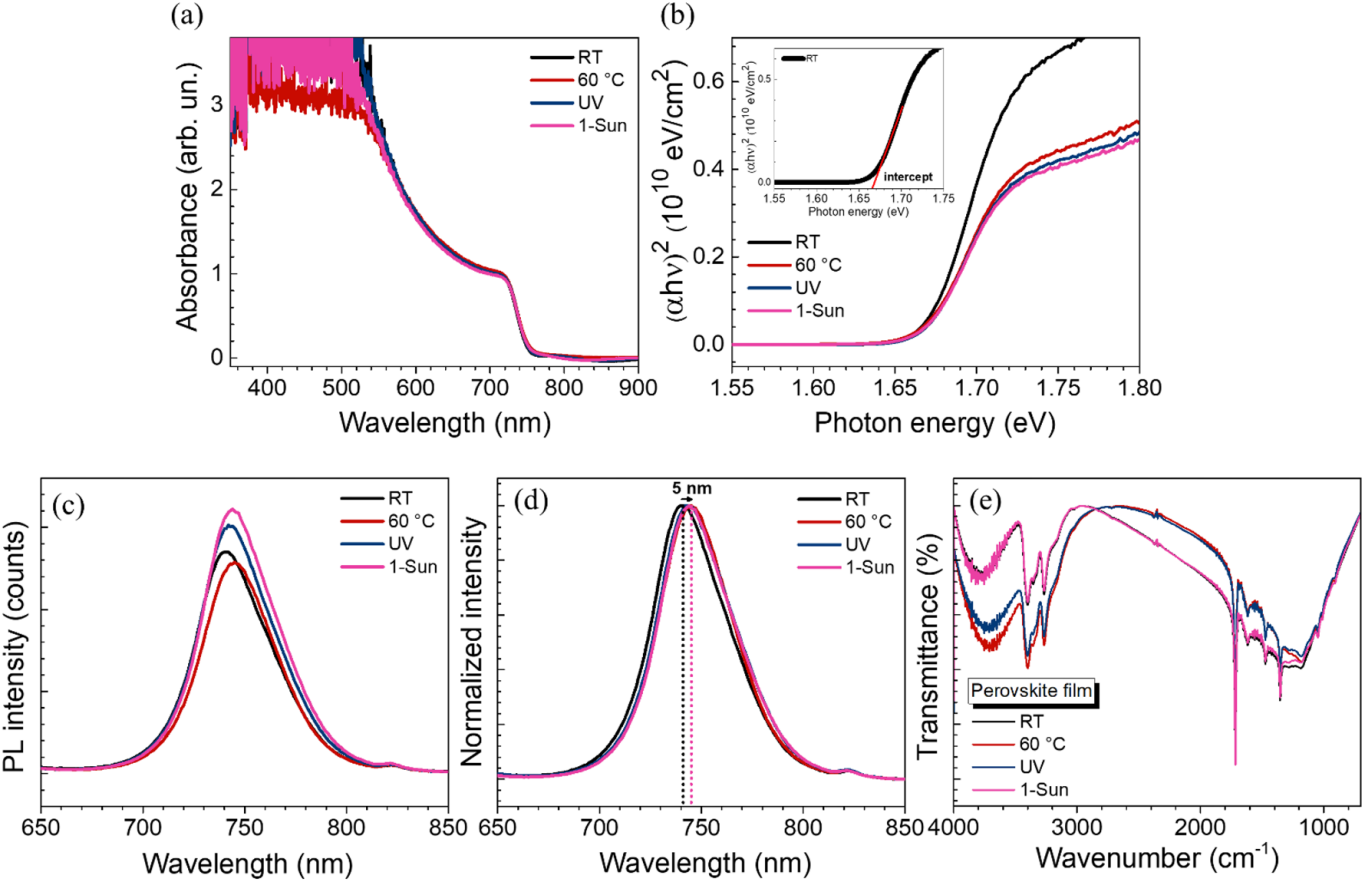
(**a**) UV–Visible absorption spectra. (**b**) Optical bandgap (E_g_). (**c**) Steady state PL emission spectra (**d**) Normalized PL emission intensity spectra. (**e**) FTIR spectra for films with RT, 60 °C, UV, and 1-Sun.

To investigate the film optical properties, absorption and emission of different perovskite films are investigated. As shown in Fig. 3a, all perovskite samples with the same thickness of 620 nm (Fig. S1c) show quite similar absorption characteristic, indicating high quality perovskite films. The optical bandgap is calculated by Tauc plot based on direct bandgap property as shown in Fig. 3b. The bandgap of our control is 1.66 eV which is in agreement with previous report^28^. However, the bandgaps of perovskite films prepared from perovskite solutions with different energy stimuli are increased from 1.66 to 1.68 eV. Therefore, photo-excited solution pathway plays an important role in enlarging the bandgap which is in agreement with the structural expansion in the unit cell parameters seen in the previously-discussed XRD results. We determined the photoluminescence (PL) emission spectra to evaluate the charge transfer dynamics of perovskite films for all conditions. The films are measured via an excitation wavelength of 500 nm as shown in Fig. 3c. Each sample was deposited on a FTO/SnO_2_ substrate. 1-Sun sample shows the strongest PL intensity, which can result from the suppressed non-radiative SRH recombination^45–47^. The PL intensities was normalized in order to assess shifting induced by different energy stimuli. The 1-Sun peak position is red-shifted by 5 nm compared to that of RT sample, confirming an increasing portion of ⍺-FAPbI_3_ within the bulk perovskite layer^48^. The FTIR measurement of different perovskite thin films are conducted in attenuated total reflection (ATR) mode in the range of 4000–400 cm^-1^ (see the FTIR spectra of perovskite precursor solution (Cs_0.05_FA_0.73_MA_0.22_Pb(I_0.77_Br_0.23_)_3_) and FAI solution in Figs. S3a–c and d–f). FTIR maps of different perovskite films are shown in Fig. S3g-j. As depicted in Fig. 3e, the transmissions which arise from the N–H stretch (around 3400 and 3250 cm^-1^) and C-N stretch (around 1000 and 900 cm^-1^) signify the presence of in FA^+^/MA^+^ in perovskite samples^49,50^. Another strong and sharp pronounced peak detected around 1700 cm^-1^ in the 1-Sun sample represents the symmetric C=N stretch that arises from more FA^+^ in the perovskite^51^, which is in agreement with our XRD and PL results.

### Dynamics after light excitation

**Figure 4:**
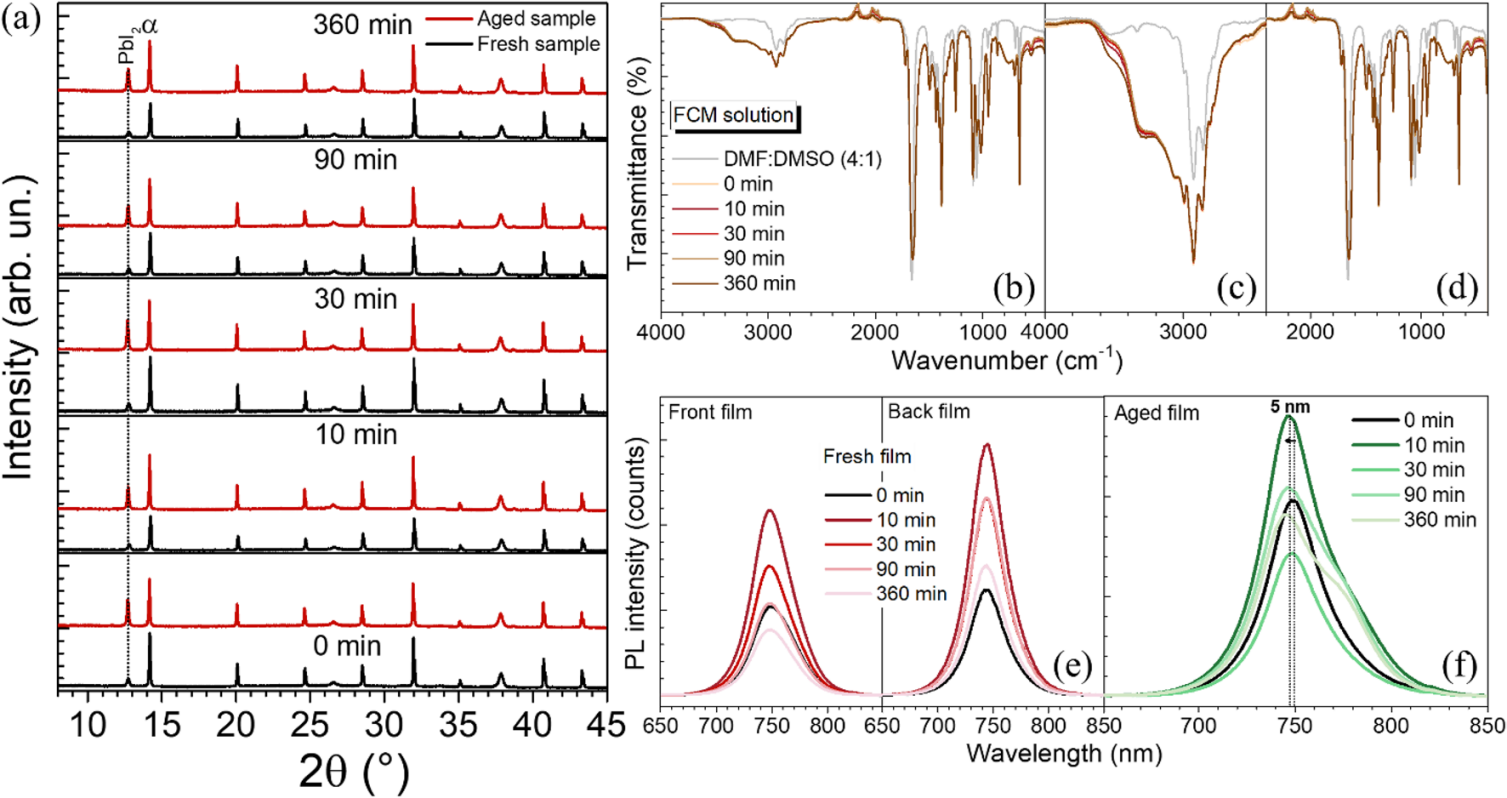
(**a**) Effective idle time duration testing after illumination (30 min under 1 sun) by keeping the solution under dark condition at different time durations of 0, 10, 30, 90, and 360 min, respectively. These solutions were used for fabricating 6 perovskite thin films, each of which was measured by XRD at 0 day (fresh, black line) and 30 days (aged, red line). Weekly XRD results are shown in Fig. S6. (110) is the perovskite characteristic peak labelled as ⍺. (**b**) FTIR spectra (**c**) Zoom-in FTIR spectra in the range of wavenumber 4000–2400 cm^-1^ and (**d**) 2400–400 cm^-1^, respectively. PL emission spectra of (**e**) fresh films and (**f**) aged films.

To study the dynamics after light excitation, excited perovskite solutions were aged and spin casted after different idle time durations (0, 10, 30, 90, and 360 min) (Fig. 2a.) The optical spectra and surface morphology of perovskite films with different idle time durations are shown in Figs. S4 and S5. As shown in Fig. 4a, the fresh films (black line) show sharper and greater peak intensities than those of the fresh films with longer idle durations especially after 30 min, indicating the improved crystallinity by light illumination pathway; the (110) peaks at 14.21$^\circ$ are gradually declined with longer idle durations after illumination. To compare films’ stability, the accelerated degradation of all perovskite films were done by storing the samples in dark humidity control dry box at relative humidity of 60% for 30 days. The red lines show XRD patterns of the aged films (after 30 days) compared to those of fresh films (0 day). After the 30 days in storage, the formation of PbI_2_ can be seen around 12.7°^32^, observed in all conditions. (see detailed stability testing results of different durations in Fig. S6).

The illuminated perovskite precursor solutions were tested by FTIR measurement as seen in Fig. 4b–d. The N–H stretch (around 3400 and 3250 cm^-1^), which represents FA^+^/MA^+^ in perovskite precursor, can be identified after the idle time duration of 10 min and further increases and slightly shifts with longer time durations (see the FTIR spectra of FAI solution in Fig. S7). PL intensity of fresh and aged perovskite films are exhibited in Fig. 4e–f, respectively. The PL intensity of the 10 min idle duration is the strongest for both font and back sides of the film, indicating lower non-radiative recombination. The peak intensities decrease after more than 10 min of no illumination, signaling decreased spontaneous radiative recombination caused by trap states on the surface and/or grain boundaries of the perovskite layer. At the same time, we observe the double peak PL spectra of perovskite thin films, indicating different phases occurring along with increasing idle time durations.

### Electrical properties

**Figure 5:**
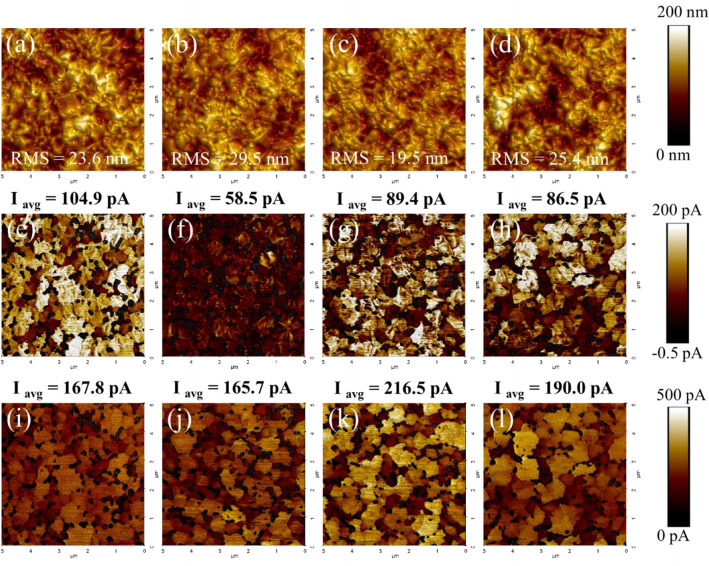
AFM topographic images and corresponding photocurrent distributions by C-AFM for different perovskite films. (**a**–**d**) Topography images for RT, 60 °C, UV, and 1-Sun samples. (**e**–**h**) The open-circuit voltage (V_oc_) mapping for RT, 60 °C, UV, and 1-Sun samples. (**i**–**l**) The short-circuit current (I_sc_) mapping for RT, 60 °C, UV, and 1-Sun samples.

The effects of energy stimuli applied to perovskite solutions on current-morphology correlation at the nanoscale were revealed by conductive atomic force microscopy (C-AFM). During the measurement, the perovskite films were excited by a white LED source (0.2 mW/cm^2^). Figure 5a–d show surface morphology and corresponding photocurrent mapping of the samples. We observe a slight increase in the root-mean-square surface roughness (RMS) from 23.6 nm (pristine sample) to 29.5 nm (60 °C sample). In contrast to photoexcited samples, RMS diminishes to 25.4 nm and dramatically drops to 19.4 nm for UV and 1-Sun, respectively. The reduction in RMS values and grain size could be due to more colloids, which form nucleation sites in those samples^27,43^. The open-circuit map (V_oc_ map) is investigated on FTO/SnO_2_/perovskite stack by setting a forward bias of 0.6 V to the AFM cantilever to simulate V_oc_ environment where the photocurrent is not far from zero as shown in Fig. 5e–h. To account for the higher traps due to the lack of the hole transport layer (HTL) in this experiment, the bias is assumed to be 0.6 V instead around 1.1 V in the actual solar device as shown in Table S1. The area with positive current in V_oc_ map represents the region where V_oc_ is more than 0.6 V, indicating perovskite surface with lower trap density^52^. Figure 5e–h show that the photocurrent decreases from 104.9 pA to 58.5 pA with the increase of the temperature from RT to 60 °C, linking to small grain and therefore more defects of the 60 °C treatment. The photocurrents are slightly reduced to 89.4 pA and 86.5 pA for UV and 1-Sun samples, agreeing with the grain size distribution trend (Fig. S8e) as observed in SEM (Fig. S8a–d). The slightly smaller grain sizes are caused by more nucleation sites stemmed from more [PbI_6_]^4−^ colloids. The short circuit current map (I_sc_ map) was done to evaluate charge conductivity pixel by pixel at zero bias under 0.2 mW/cm^2^ irradiation as shown in Fig. 5i–l. Interestingly, both UV and 1 sun illuminated samples exhibit significantly higher photocurrents compared to those of RT and 60 °C samples, indicating superior charge transport^53^ on the grain surfaces due to less iodide vacancies from more [PbI_6_]^4−^ population; however, lower photocurrents are observed at the grain boundaries in all conditions in agreement with the darker and therefore higher defect areas in the V_oc_ map. The poor photocurrent for the 60 °C sample can be explained by high trap density and small morphological grains, as observed from SEM (Fig. S8a-d) along with the high root mean square value of roughness (RMS) in Fig. 5b, which relates faster crystallization at elevated temperature.

### Solar cell device performances

**Figure 6:**
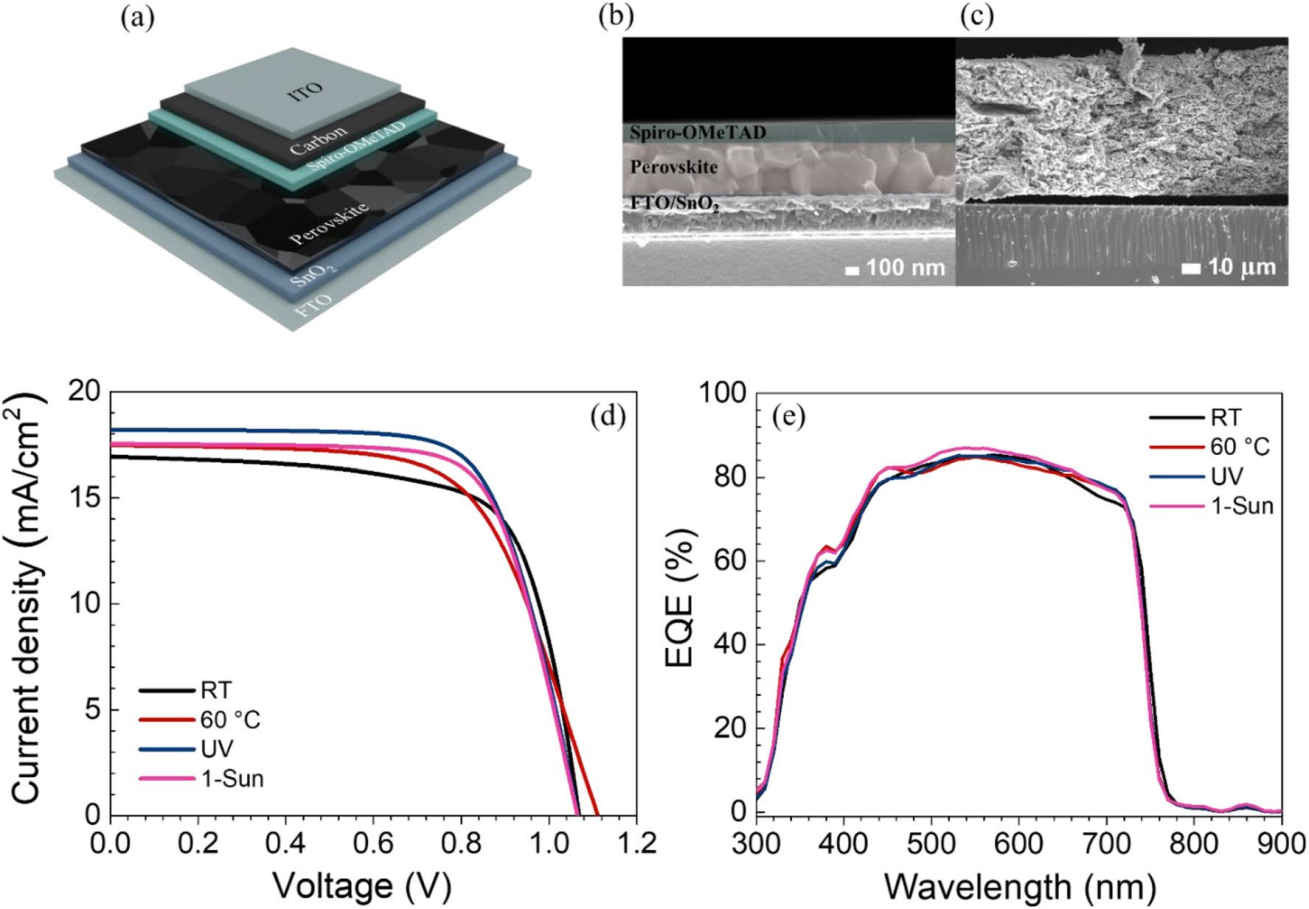
(**a**) Illustration of carbon-based perovskite solar cell. (**b**) Cross-sectional SEM images of perovskite solar cell with (**c**) carbon-based back-electrode. (**d**) J-V curves of different perovskite solar cells with carbon electrode. (**e**) EQE spectra.

The two light sources are 1 sun irradiation (AM1.5G, 100 mW/cm^2^) and LED (illuminance: 1000 lux, 0.31 mW/cm^2^) to investigate the incident light-dependent photovoltaic performances for outdoor and indoor usages. The average photovoltaic parameters, which include V_oc_, J_sc_, FF, and PCE are summarized in Table S1. All devices were fabricated using low-cost carbon electrode. The schematic of n-i-p device configuration (FTO/SnO_2_/perovskite/spiro-OMeTAD/carbon/ITO) is shown in Fig. 6a. Besides, cross-sectional SEM images of the carbon-based architecture is shown in Fig. 6b, showing large grain, smooth, and dense layers for the 1-Sun sample. Figure 6c illustrates the carbon electrode in the device. Figure 6d shows J-V characteristics of the best devices from RT, 60 °C, UV, and 1-Sun conditions. The PCE of UV and 1-Sun devices are superior to those of RT and 60 °C conditions with the PCEs of 13.62% and 13.25%, respectively. The PCE performances under low light condition show a similar trend with 1 sun illumination. The J-V curves of the best devices under low light condition and an irradiance spectrum of indoor light source (cool daylight LED) are shown in Fig. S9a,b. Moreover, the highest and second highest current density (J_sc_) of 18.2 mA/cm^2^ and 17.5 mA/cm^2^ are observed in UV and 1 sun treated devices, respectively. The clearly improved current density is due to reduced vacancy defects from more full-coordination iodoplumbates^27^. These results are in agreement with the relatively good photocurrents confirmed by C-AFM as shown in Fig. 5i–l. The ratio of generated electrons to given photons at a specific wavelength of light excitation were identified; the external quantum efficiency (EQE) spectra of the best PSC devices are displayed in Fig. 6e. The higher EQE results for 1-Sun and UV samples are consistent with the J_sc_ from J-V performances and the photocurrent results in the I_sc_ maps from C-AFM. We also applied our external stimuli methods to other popular types of perovskites which are MAPbI_3_, Cs_0.17_FA_0.83_PbI_2.49_Br_0.51_ (CsFA), and Cs_0.05_FA_0.81_MA_0.14_PbI_2.55_Br_0.45_ (CsFAMA). The solar cell performances under 1 sun illumination and low light condition are shown in Figs. S10 and S11, respectively. These results indicate that the chemistry of perovskite precursors also plays an important role with different responses to the external stimuli.

**Figure 7:**
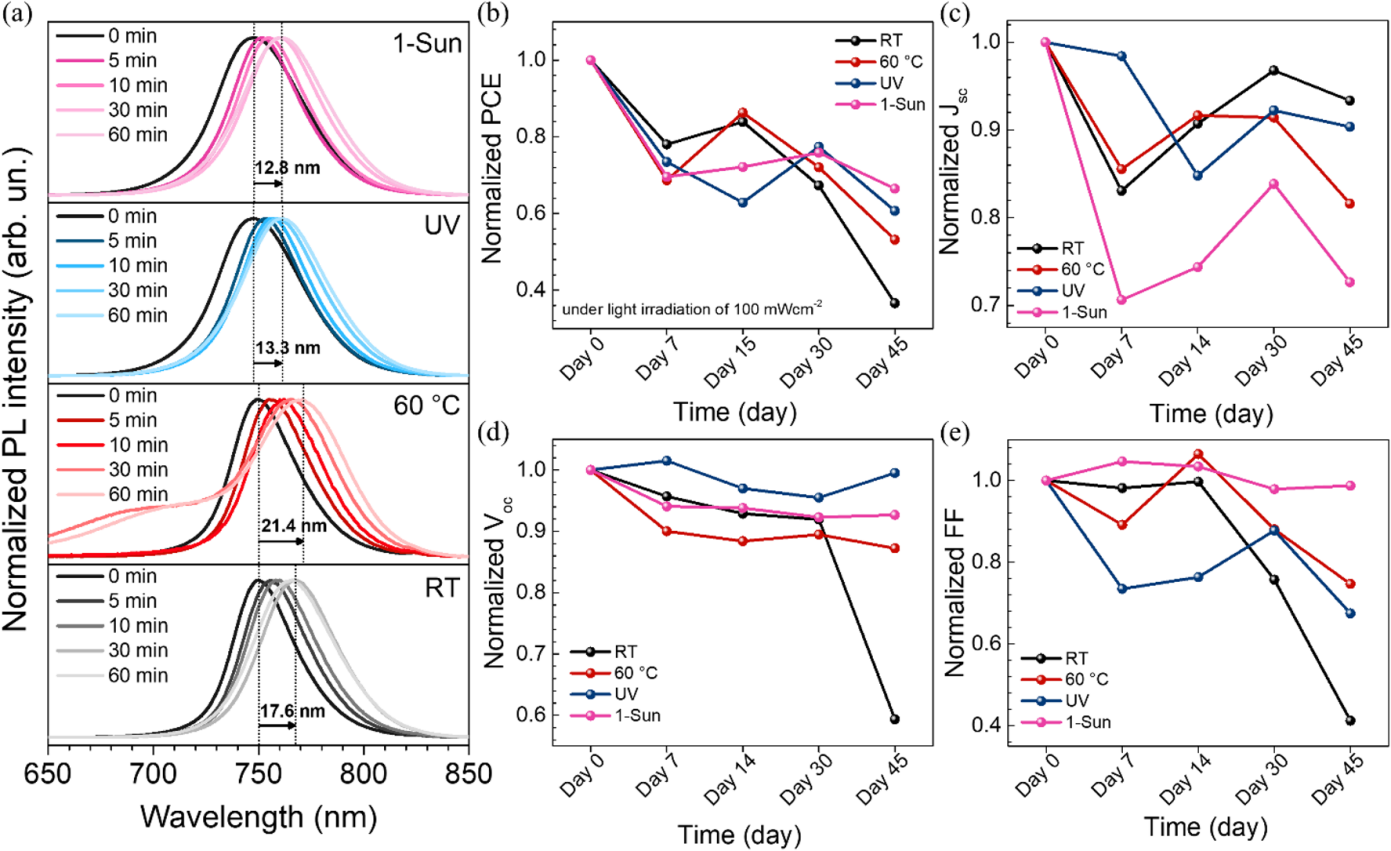
(**a**) Photoluminescence (PL) spectra of perovskite films exposed to AM1.5G. (**b**–**e**) Long-term stability of the unsealed devices based on the pristine and different external stimuli-treated perovskites stored in dark condition with humidity of 40–60%.

The problem of wide-bandgap perovskite with high bromine is phase instability. The phase segregation between iodine- and bromide-rich regions in the perovskite layer under light illumination is observed through in PL measurement as shown in Fig. 7a. In fact, the iodine-rich region can lead to charge recombination, which can reduce the V_oc_ and FF under AM1.5G irradiation^54,55^. The PL shift began within 5 min of 1 sun irradiation for all perovskite films. After 60 min of 1 sun illumination, the 1-Sun and UV films exhibited smaller photoluminescence peak shifts compared to those of control and 60 °C conditions, which suggest higher phase stability. We also investigated the stability of perovskite solar cells (PSCs), which is a crucial issue for commercialization. Figure 7b–e shows the normalized PCE of the unsealed PSCs under 1 sun illumination. After 45 days, the PCE decreased along with other parameters. However, the PSCs subjected to 1 sun and UV irradiation show smaller changes in PCE, indicating improved device stability due to the photoenergy pathway.

**Figure 8:**
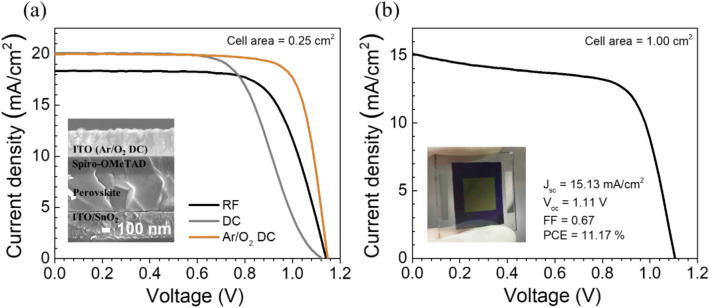
(**a**) J-V curves of semi-transparent perovskite solar cells with different ITO electrodes (cell area = 0.25 cm^2^). The inset shows the cross-sectional SEM of the device architecture. (**b**) J-V curve of semi-transparent perovskite solar cell with the cell area of 1.00 cm^2^. The inset shows the actual solar device with the active area 1.00 cm^2^.

To demonstrate the potential as top cell material for tandem architecture due to its high current, the 1 sun-treated precursor was used to make perovskite cells with transparent electrodes (ITO), which were fabricated by different processes, radio frequency (RF), direct current (DC), and Ar/O_2_ direct current (Ar/O_2_ DC) magnetron sputtering. The samples are labelled as RF, DC, and Ar/O_2_ DC devices, respectively. The J-V characteristic was performed with an active area of 0.25 cm^2^; the inset of Fig. 8a show cross-sectional SEM of the device architecture. As shown in Fig. 8a, different ITO electrodes mainly affect FF and J_sc_, while V_oc_ remains comparable to that of the carbon electrode. The solar cell performances are shown in Table S2. The Ar/O_2_ DC device shows lowest R_s_ of 5.59 Ωcm^2^, which is caused by good contact between ITO and HTL, leading to the excellent FF value of 0.78 and the PCE of 17.9%. However, the poor R_s_’s of 10.42 Ωcm^2^ from RF and 61.35 Ωcm^2^ from DC are revealed, causing low FF and PCE. Furthermore, the performance of larger scale device from Ar/O_2_ DC process with an active area of 1.00 cm^2^ was also measured as shown in Fig. 8b. The large-scale device shows the PCE of 11.2% with J_sc_ of 15.13 mA/cm^2^, V_oc_ of 1.11 V, and FF of 0.67. The statistics are shown in Table S2.

## Conclusions

In this work, we modified the iodoplumbate equilibrium in a triple cation perovskite precursor system to investigate the correlation between iodoplumbate species within the perovskite solutions under different external stimuli. 1 sun and UV irradiation can easily affect the equilibrium without any additives, leading to great conversion to high-valent iodoplumbate species in perovskite precursor solutions. For the illuminated perovskite precursors, the crystallinity is greatly enhanced along with smooth morphology and reduced defect density. The resulting films from UV and 1-Sun precursor solutions have improved photocurrents as seen from C-AFM and solar cell performances due to reduced donor defects such as iodide vacancies from high-valent iodoplumbate species. With 1 sun treatment, the PCEs of 13.6% and 17.9% (cell area of 0.25 cm^2^) were obtained under 1 sun illumination by using low-cost carbon and ITO as the electrodes, respectively. The J_sc_ is significantly better than those of the untreated and the thermal-treated perovskite solutions. Moreover, the same concept was further demonstrated with a large-scale semi-transparent device having transparent electrode and the cell area of 1.00 cm^2^, yielding the PCE of 11.2% along with the J_sc_ of 15.13 mA/cm^2^, the V_oc_ of 1.11 V, and the FF of 0.67. Our results suggest that precise control of chemical environment of iodoplumbates in perovskite precursor solution by light treatment is critical for fabricating highly efficient PSCs. While the process was mainly tested on a wide-bandgap material for silicon/perovskite tandem solar cell technology and low light photovoltaic, this similar approach is likely useful for other perovskite compositions for various optoelectronic applications.

## Supplementary Information

Supplementary Information.

## Data Availability

The datasets used and/or analysed during the current study available from the corresponding author on reasonable request.
