# Supplementary material for: Photoexcitation of perovskite precursor solution to induce high-valent iodoplumbate species for wide bandgap perovskite solar cells with enhanced photocurrent
Source: Sci Rep. 2023 Apr 14;13:6125. doi: 10.1038/s41598-023-32468-w (PMC10104826; doi:10.1038/s41598-023-32468-w)
Supplement: Supplementary file 1 — Supplementary Information. [file 41598_2023_32468_MOESM1_ESM.docx]

**Photoexcitation of Perovskite Precursor Solution to Induce High-valent Iodoplumbate Species for Wide Bandgap Perovskite Solar Cells with Enhanced Photocurrent**

Atittaya Naikaew^1,2^, Taweewat Krajangsang^1^, Ladda Srathongsian^2^, Chaowaphat Seriwattanachai^2^, Patawee Sakata^2^, Supavudh Burimart^2^, Kanyanee Sanglee^1^, Kittikhun Khotmungkhun^2^, Pipat Ruankham^3^, Suwat Romphosri^2^, Amornrat Limmanee^1^, and Pongsakorn Kanjanaboos^2,4*^

^1^ National Energy Technology Center (ENTEC), National Science and Technology Development Agency, Pathum Thani 12120, Thailand

^2^ School of Materials Science and Innovation, Faculty of Science, Mahidol University, Nakhon Pathom 73170, Thailand

^3^ Department of Physics and Materials Science, Faculty of Science, Chiang Mai University, Chiang Mai 50200, Thailand

^4^ Center of Excellence for Innovation in Chemistry (PERCH-CIC), Ministry of Higher Education, Science, Research and Innovation, Bangkok 10400, Thailand

*pongsakorn.kan@mahidol.edu

Supplementary Information

**Materials**

Perovskite precursors were comprised of lead(II) iodide (PbI_2_; 99.99%, trace metals basis), lead(II) bromide (PbBr_2_; >98%), formamidinium iodide (FAI; ≥99%), methylammonium bromide (MABr; 98%), cesium iodide (CsI; 99.9%, trace metals basis), methylammonium iodide (MAI; 99.99%,), anhydrous *N, N*-dimethylformamide (DMF; 99.8%v/v), anhydrous dimethyl sulfoxide (DMSO; 99%v/v), anhydrous γ-Butyrolactone (GBL; ≥99%,), anisole (anhydrous; 99.7%), anhydrous toluene (TLE; 99.85%), and anhydrous chlorobenzene (CB; 99.8%). Electron and hole transport precursors are comprised of tin(II) chloride dihydrate (SnCl_2_·2H_2_O; 99.999%), anhydrous ethanol (ethanol; 99.5%v/v), 4-tert-butylpyridine (tBP), lithium bis-(trifluoromethanesulfonyl) imide (Li-TFSI), acetonitrile (anhydrous; 99.8%), and spiro-OMeTAD. PbI_2_ and PbBr_2_ perovskite precursors were purchased from TCI CO., LTD. FAI, MABr, MAI, TEC 15 FTO glass substrates were purchased from Greatcell Solar Materials Pty Ltd. CsI, DMF, DMSO, GBL, CB, anisole, EtOH, SnCl_2_·2H_2_O, tBP, Li-TFSI, acetonitrile, and spiro-OMeTAD were purchased from Sigma-Aldrich. TLE was purchased from ACROS Organics. TEC 15 ITO glass substrates were purchased from Luminescence Technology Corp. Carbon paste Jelcon CH-8 (low-resistance carbon paste with excellent performance) was from Jujo Chemical CO., LTD.

**Perovskite precursor solutions**

For each 1 ml of the wide bandgap triple cation perovskite Cs_0.05_FA_0.73_MA_0.22_Pb(I_0.77_Br_0.23_)_3_, 1.5 M of PbI_2_ and PbBr_2_ in DMF:DMSO of 4:1 v/v were first prepared by stirring overnight at 60 °C while 1.5 M CsI was dissolved in DMSO and stirred overnight at room temperature as stock solutions (stock solutions were prepared one day before mixing). To obtain the FAPbI_3_ and MAPbBr_3_, each stock solution of PbI_2_ and PbBr_2_ was then added into 198.6 mg FAI and 38.6 mg MABr with 9 mol% PbX_2_ excess (X = I or Br), respectively. The double cation perovskite was obtained by mixing FAPbI_3_ and MAPbBr_3_ in 77:23 volume ratio. Finally, 5 vol% of CsI stock solution was added to double cation perovskite to obtain the triple cation perovskite precursor. Then, stirred until completely dissolved and filtered with 0.22 μm PTFE CNW syringe filter.

We have investigated other types of perovskites such as MAPbI_3_, Cs_0.17_FA_0.83_PbI_2.49_Br_0.51_ (CsFA), and Cs_0.05_FA_0.81_MA_0.14_PbI_2.55_Br_0.45_ (CsFAMA). For MAPbI_3_, in-stoichiometric MAPbI_3_ precursor solution was carried out by 1:1 mole ratio of PbI_2_ and MAI in a 7:3 v/v mixture of GBL and DMSO (1.5 M). The precursor solution was stirred at 70 °C for 30 min on a hotplate to confirm that the mixed precursor was completely dissolved. The solution was then filtered with a poly(tetrafluoroethylene) (PTFE) syringe filter (0.22 µm) before film fabrication. Then, the solution was spin-coated onto a substrate at 500 rpm for 10 s with an acceleration of 250 rpm/s, followed the second speed protocol at 5,000 rpm for 60 s with the acceleration of 1,500 rpm/s. The 120 µl anhydrous TLE was then applied at 30^th^ s as an anti-solvent. Finally, the film was annealed at 100 °C for 15 min.

CsFA perovskite at 1.3 M could be obtained by following the procedure described in the previous work.^1^ To obtain the CsFA, FAI (188.3 mg), CsI (56.4 mg), PbI_2_ (478.3 mg), and PbBr_2_ (121.7 mg) were mixed into DMF and DMSO in a volume ratio of 4:1 for 1 ml of the precursor solution. The perovskite layer was done inside a N_2_-filled glovebox. The perovskite film was successively spun onto the FTO/SnO_2_ layer at 1,000 rpm for 13 s with the acceleration of 500 rpm/s and then at 4,000 rpm for 30 s with the acceleration of 2,000 rpm/s. Just five seconds before the end of the spin-coating process, 180 μl of anisole was rapidly dropped onto the perovskite precursor as an anti-solvent. The sample was then annealed on a hot plate at 130 °C for 15 min.

To prepare the CsFAMA perovskite precursor solution^2^, To obtain the 1.2 M (1 ml) mixture, the precursor solution is prepared by combining FAI (171.2 mg), PbBr_2_ (68.0 mg), PbI_2_ (467.0 mg), MABr (19.5 mg), and CsI (16.4 mg) in DMF:DMSO of 4:1 v/v within a N_2_-filled glovebox. To deposit the perovskite films onto the FTO/SnO_2_ substrates, 50 µl of the perovskite precursor was spin-coated at a spin speed of 1,000 rpm for 10 s with the acceleration of 500 rpm/s, followed by the second spin speed protocol of 4,000 rpm for 30 s with the acceleration of 2,000 rpm/s. At the 21^st^ s from the first step, 100 µl of anhydrous CB was added as an anti-solvent. Finally, the film was annealed at 100 °C for 15 min.

**Supplementary Figures**

**
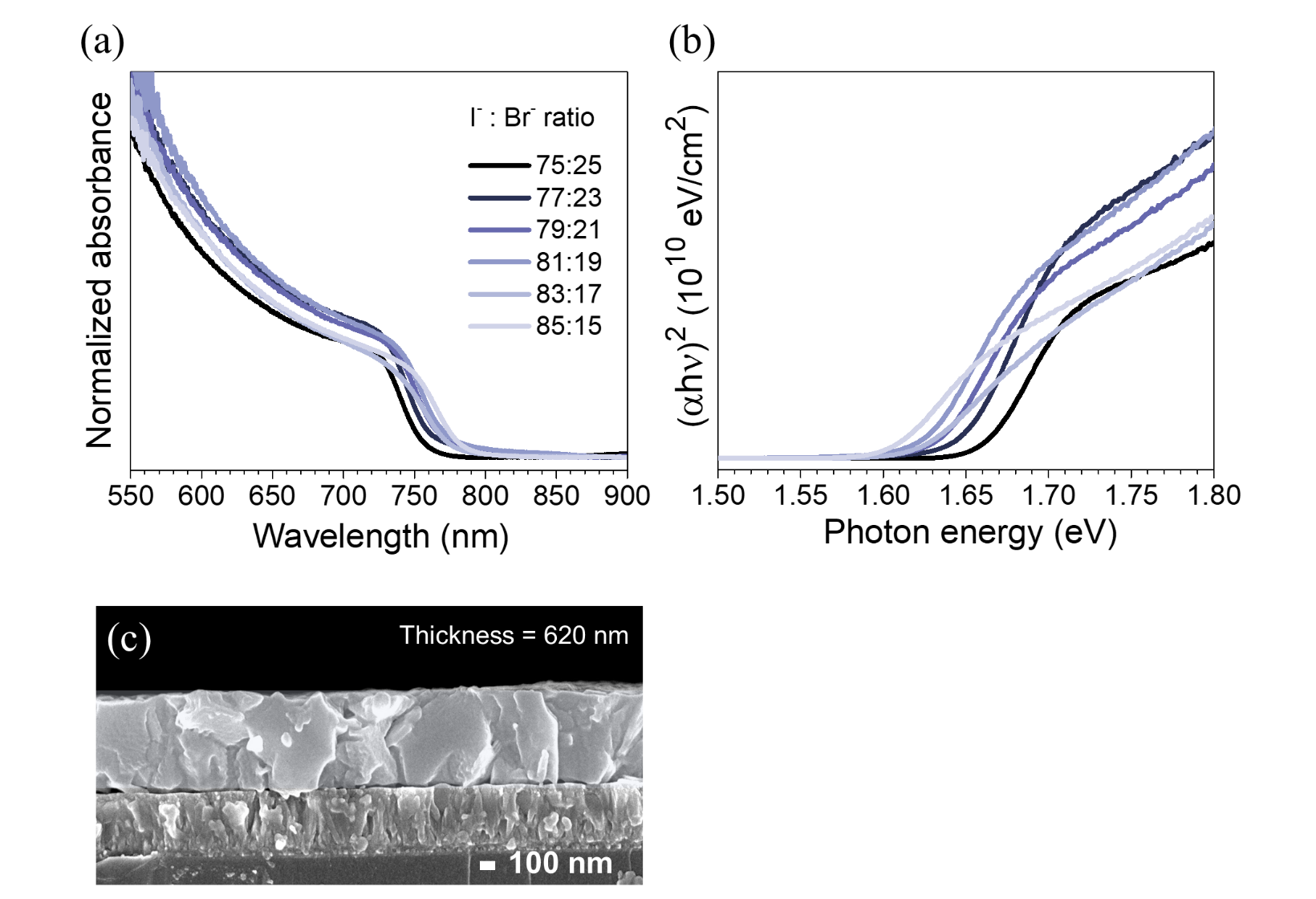
**

**Figure S1.** (a) UV-Vis spectra. (b) Optical bandgap (E_g_) of perovskite films with different I^-^ and Br^-^ ratios. (c) SEM cross-section of perovskite film.


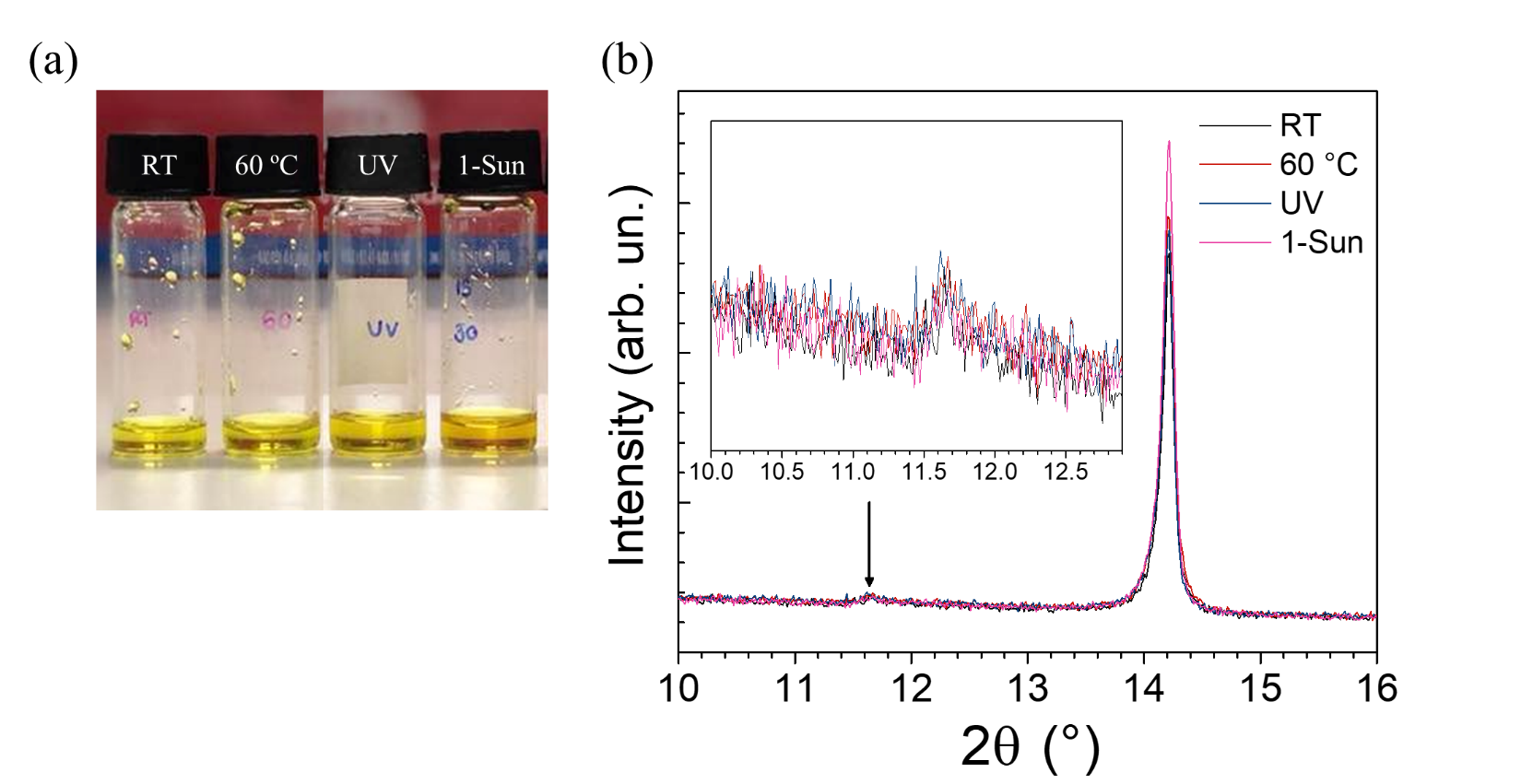


**Figure S2.** (a) Photograph of perovskite precursor solutions. (b) Narrow view of perovskite crystal planes measured between 10-16°. Active perovskite phase (⍺) and inactive perovskite phase (𝛿) are found at 14.21° and 11.57°, respectively.

**
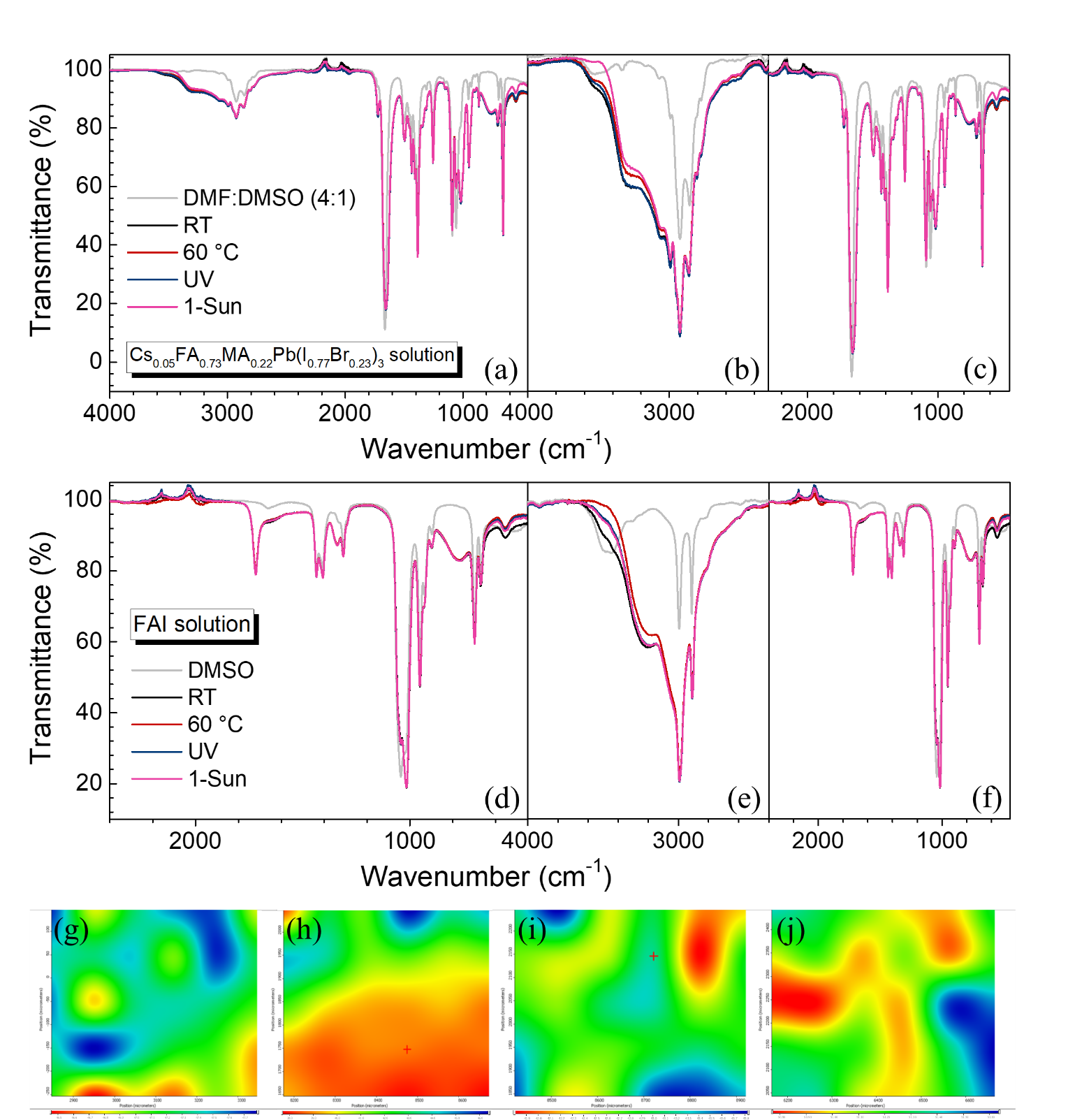
**

**Figure S3.** FTIR spectra of (a) different perovskite precursor solutions with different stimuli. Narrow view of perovskite solutions’ spectra between (b) 4,000-2,400 cm^-1^ and (c) 2,400-400 cm^-1^. (d) FTIR spectra of FAI solution with different stimuli. Narrow view of FAI solutions’ spectra between (b) 4,000-2,400 cm^-1^ and (c) 2,400-400 cm^-1^. (g-j) FTIR mapping of different perovskite films (RT, 60 °C, UV, and 1-Sun).

**Figure S4.** Optical spectra of perovskite films with different idle time durations of 0, 10, 30, 90, and 360 min after 1 sun treatment.

**
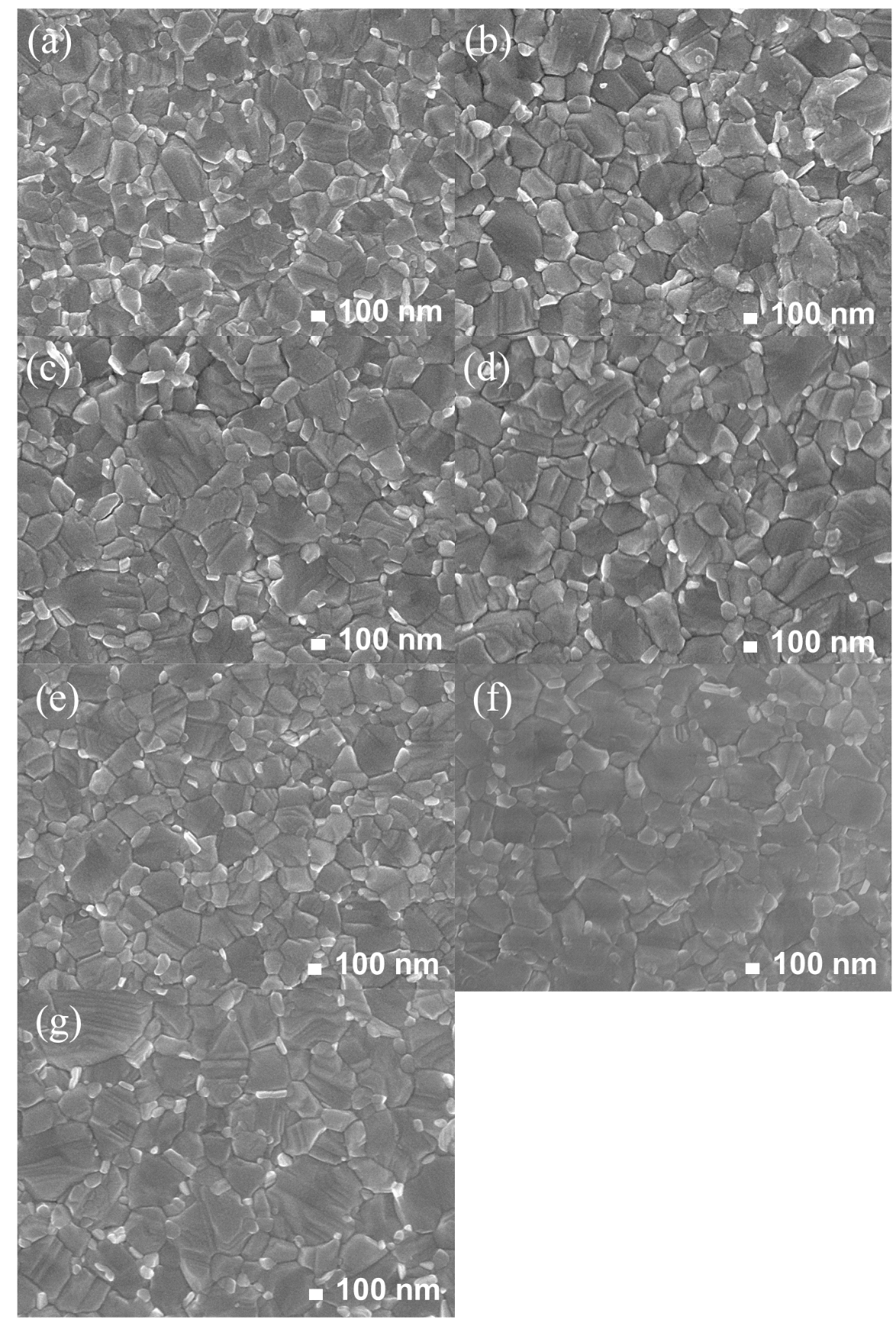
**

**Figure S5.** SEM morphology of perovskite films with different idle time durations of (a) 0, (b) 3, (c) 10, (d) 30, (e) 90, (f) 240, and (g) 360 min after 1 sun treatment.

**
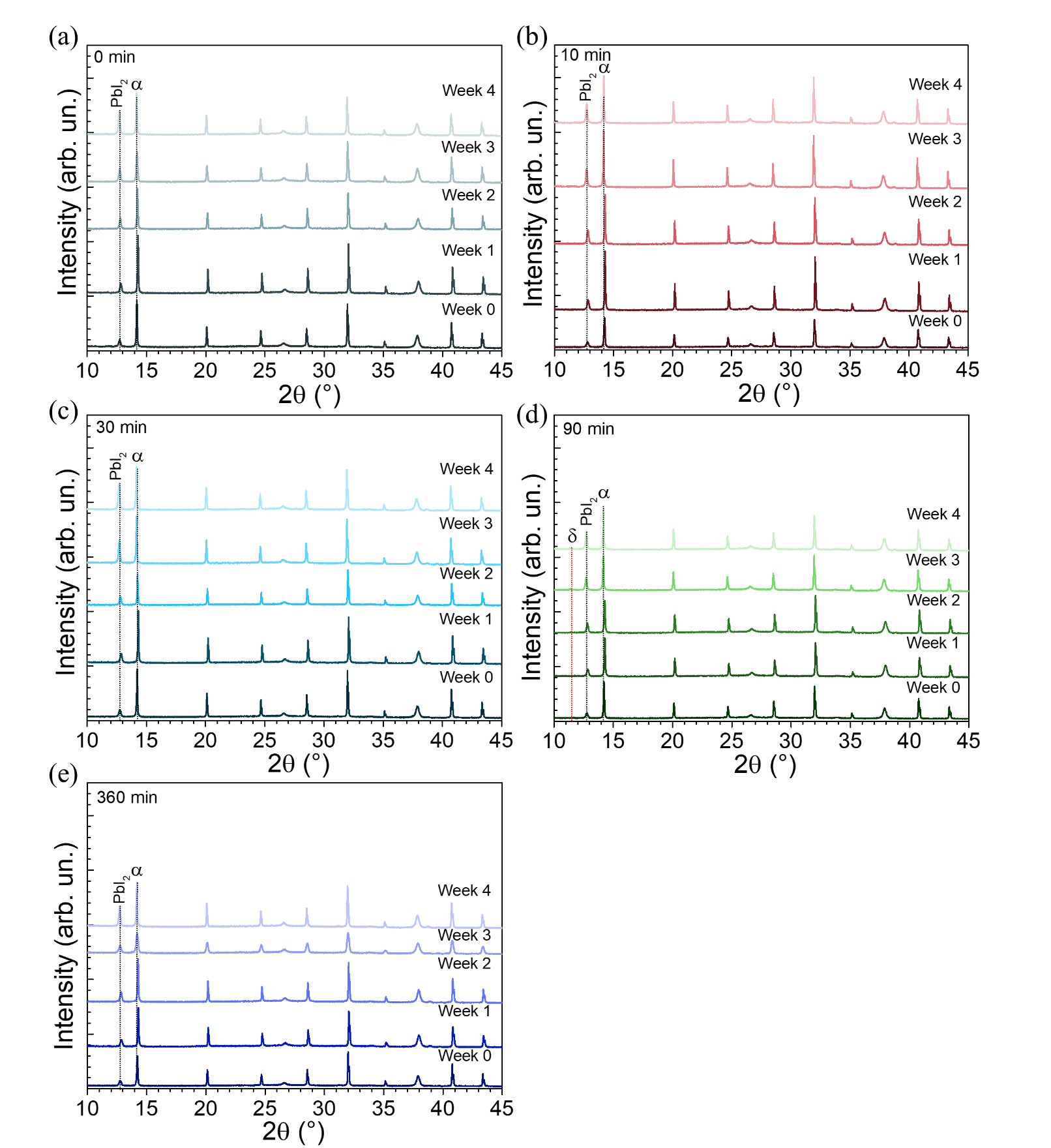
**

**Figure S6.** Stability testing of perovskite films with different idle time durations of (a) 0, (b) 10, (c) 30, (d) 90, and (e) 360 min after 1 sun treatment.

**
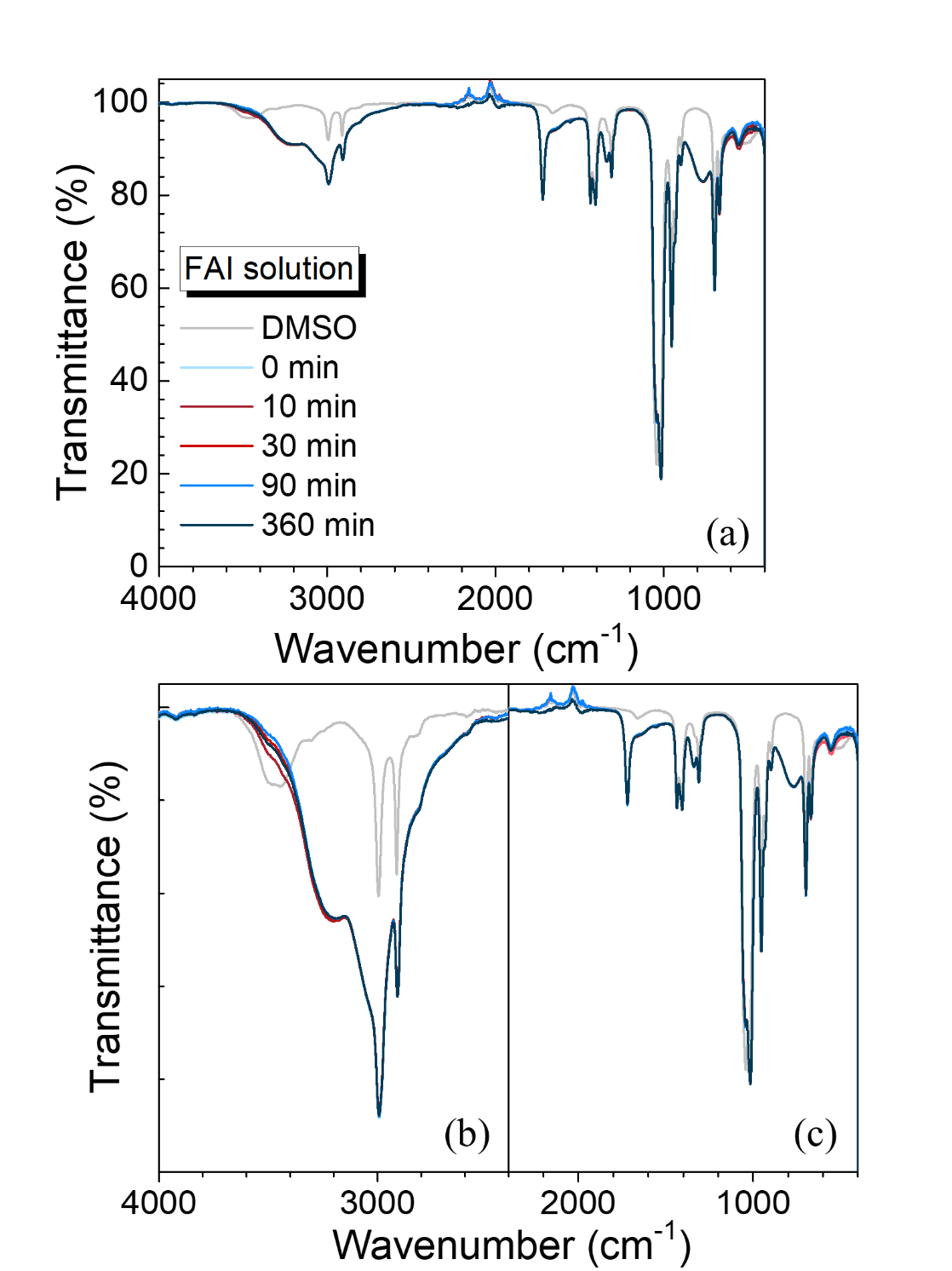
**

**Figure S7.** (a) FTIR spectra of different FAI solutions with different idle time durations of 0, 10, 30, 90, and 360 min after 1 sun treatment. Narrow view of FAI solutions’ spectra within (b) 4,000-2,400 cm^-1^ and (c) 2,400-400 cm^-1^.

**Table S1**. The photovoltaic parameters of perovskite solar devices with carbon-based back-electrode under 1 sun (100 mW/cm^2^) and low light illumination (1000 lux).

| **Light source** | **Condition** | **V_oc_ (V)** | **J_sc_ (mA/cm^2^)** | **FF** | **PCE (%)** | **J_sc, EQE_ (mA/cm^2^)** |
| --- | --- | --- | --- | --- | --- | --- |
| AM1.5G  1000 lux | RT | 1.07  0.90 | 16.93  0.117 | 0.69  0.71 | 12.60  24.05 | 18.27 |
| AM1.5G  1000 lux | 60 °C | 1.11  0.90 | 17.50  0.116 | 0.64  0.74 | 12.35  24.90 | 18.53 |
| AM1.5G  1000 lux | UV | 1.07  0.86 | 18.20  0.120 | 0.70  0.74 | 13.62  24.69 | 18.71 |
| AM1.5G  1000 lux | 1-Sun | 1.07  0.86 | 17.53  0.128 | 0.71  0.71 | 13.25  25.50 | 18.97 |


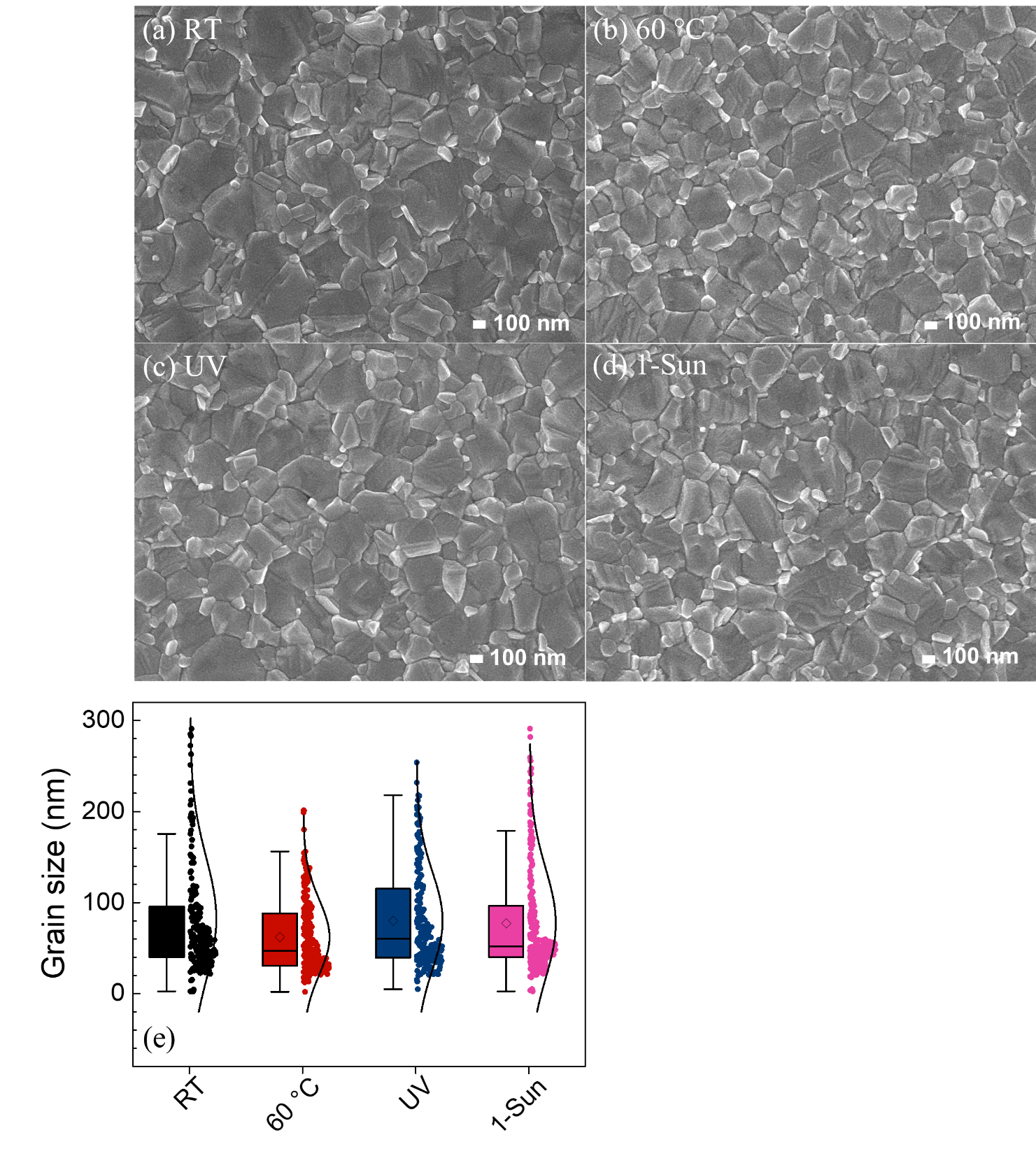


**Figure S8.** Film surface morphologies of (a) RT, (b) 60 °C, (c) UV, and (d) 1-Sun. (e) Grain size distribution of perovskite thin films.

**
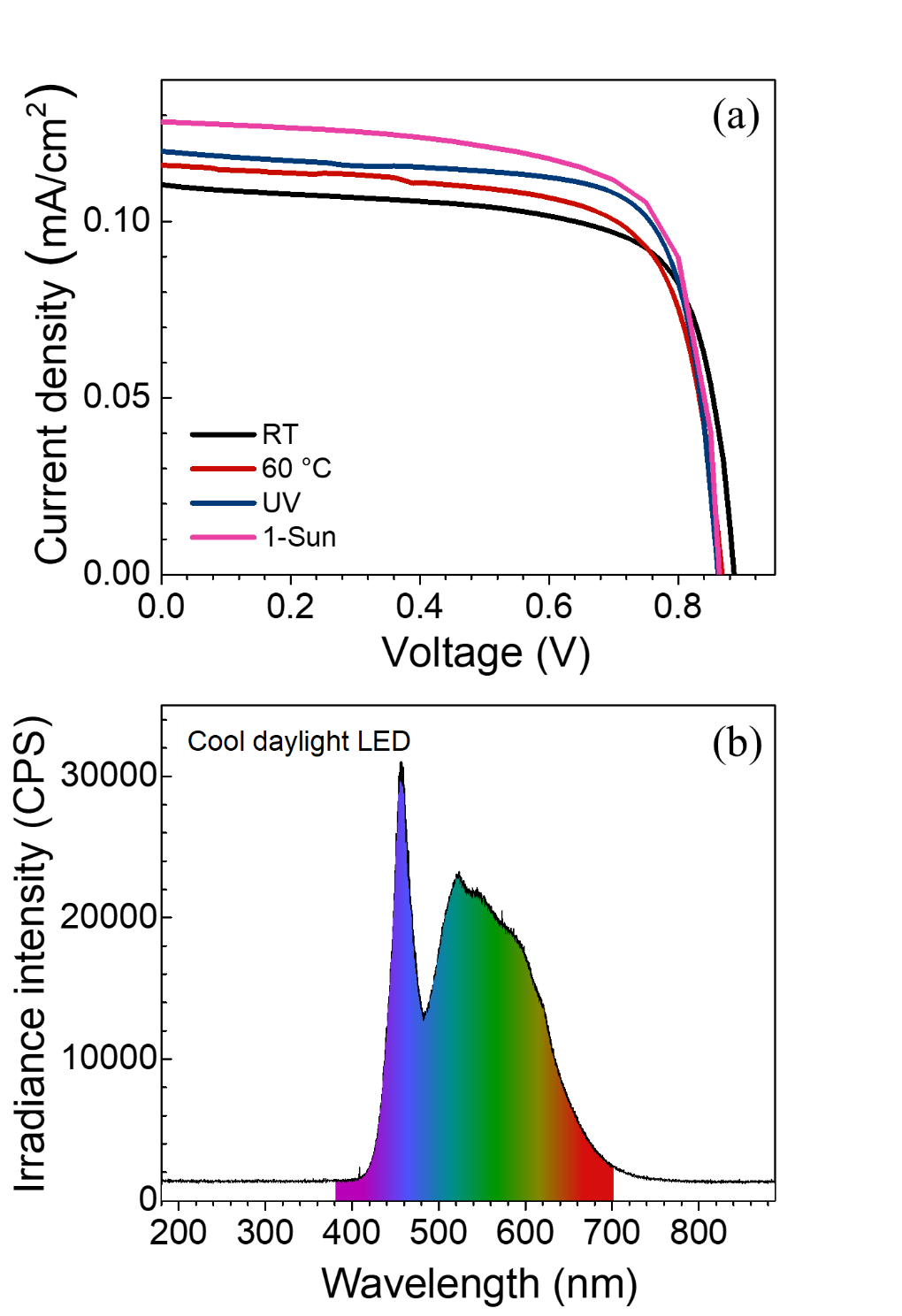
**

**Figure S9.** (a) J-V curves of different perovskite solar cells with carbon electrode under LED light illumination (1000 lux, 0.31 mW/cm^2^). (b) Irradiance intensity of indoor light source (Philips cool daylight LED, 4W).

**
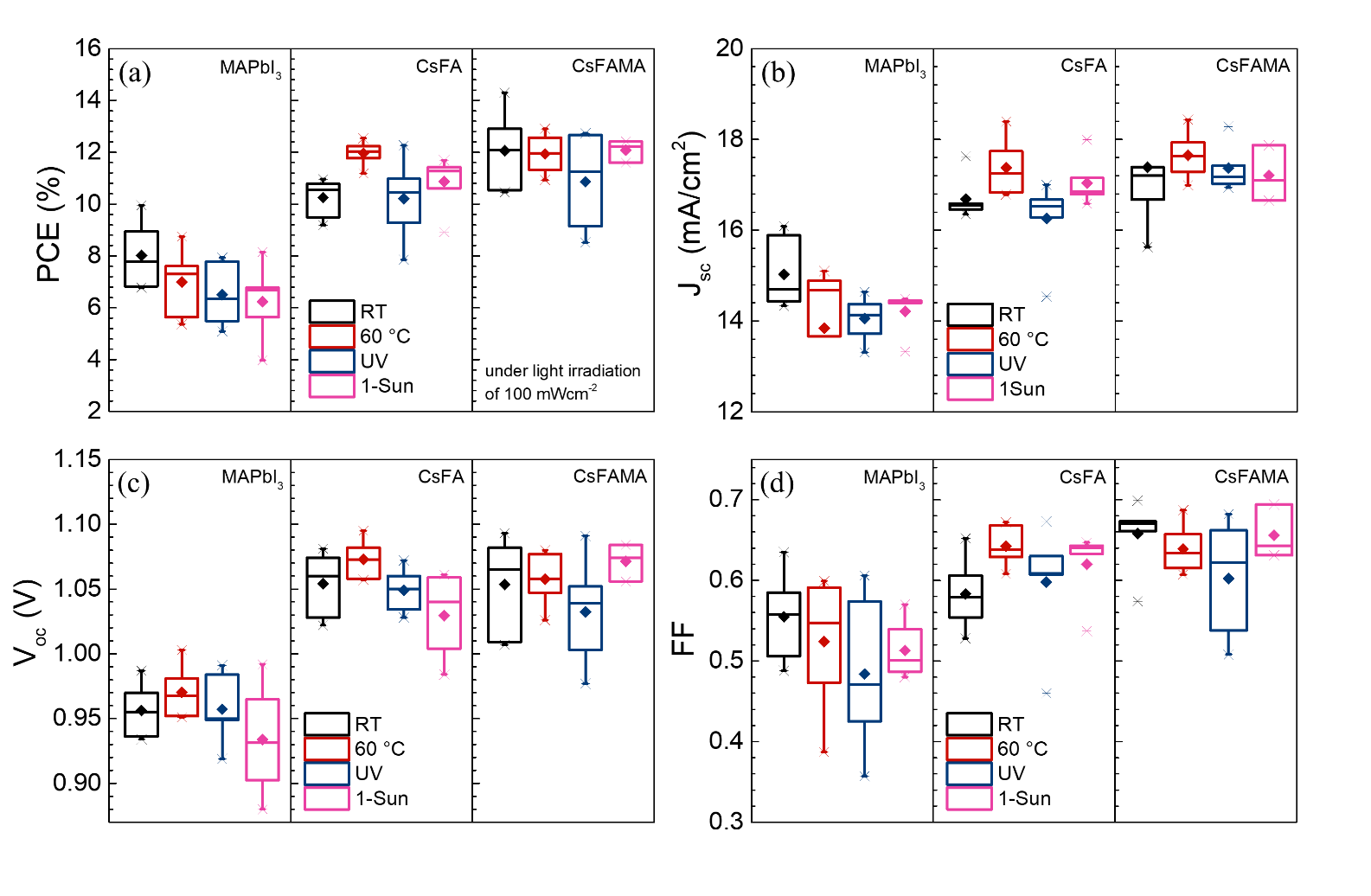
**

**Figure S10.** (a) Box plot of PCE, (b) short circuit current density, (c) open circuit voltage, and fill factor of MAPbI_3_, Cs_0.17_FA_0.83_PbI_2.49_Br_0.51_ (CsFA), and Cs_0.05_FA_0.81_MA_0.14_PbI_2.55_Br_0.45_ (CsFAMA) devices under 1 sun illumination.

**
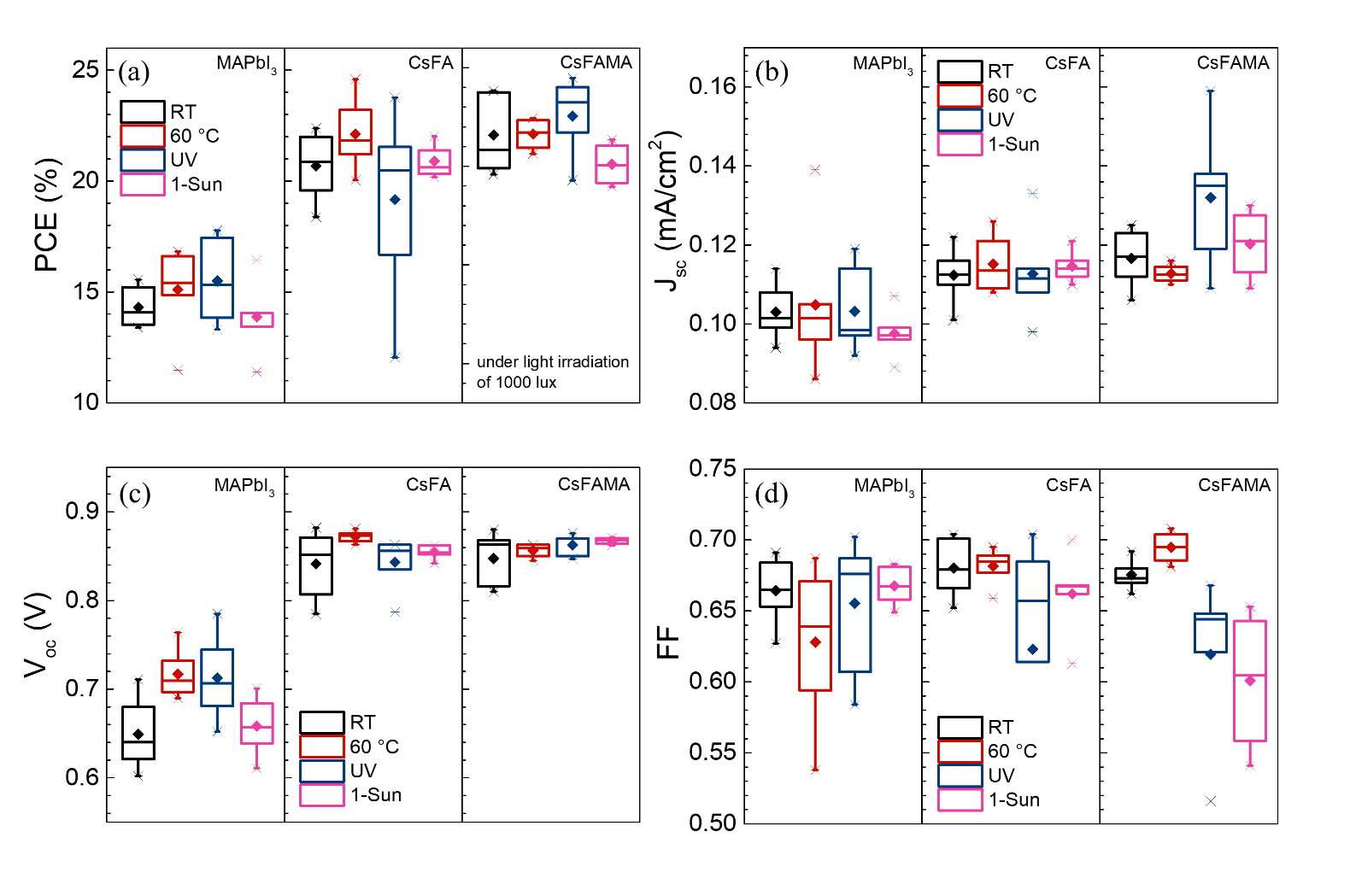
**

**Figure S11.** (a) Box plot of PCE, (b) short circuit current density, (c) open circuit voltage, and fill factor of MAPbI_3_, Cs_0.17_FA_0.83_PbI_2.49_Br_0.51_ (CsFA), and Cs_0.05_FA_0.81_MA_0.14_PbI_2.55_Br_0.45_ (CsFAMA) devices under 1,000 lux LED illumination.

**Table S2**. The photovoltaic parameters of perovskite solar devices with different ITO-based back-electrodes under 1 sun illumination (100 mW/cm^2^).

| **Condition** | **Cell area (cm^2^)** | **Sample** | **V_oc_ (V)** | **J_sc_ (mA/cm^2^)** | **FF** | **PCE (%)** | **R_sh_**  **(ohm.cm^2^)** | **R_s_**  **(ohm.cm^2^)** |
| --- | --- | --- | --- | --- | --- | --- | --- | --- |
| RF | 0.25 | 1 | 1.11 | 16.62 | 0.64 | 11.77 | 69.44 | 15.92 |
| RF | 0.25 | 2 | 1.16 | 15.96 | 0.64 | 11.76 | 555.56 | 16.95 |
| RF | 0.25 | 3 | 1.14 | 18.32 | 0.70 | 14.65 | 1,666.67 | 10.42 |
| RF | 0.25 | 4 | 1.14 | 18.39 | 0.67 | 14.05 | 1,666.67 | 10.92 |
| Average | 0.25 |  | 1.14 | 17.32 | 0.66 | 13.06 | 989.58 | 13.55 |
| DC | 0.25 | 1 | 1.08 | 18.76 | 0.55 | 11.13 | 1,666.67 | 80.00 |
| DC | 0.25 | 2 | 1.12 | 16.83 | 0.58 | 11.01 | 227.27 | 57.14 |
| DC | 0.25 | 3 | 1.13 | 20.08 | 0.61 | 14.05 | 833.33 | 61.35 |
| DC | 0.25 | 4 | 1.12 | 19.79 | 0.61 | 13.55 | 1,666.67 | 49.75 |
| Average | 0.25 |  | 1.11 | 18.86 | 0.59 | 12.44 | 1,098.48 | 62.06 |
| Ar/O_2_ DC | 0.25 | 1 | 1.08 | 19.94 | 0.73 | 15.80 | 1,428.57 | 8.48 |
| Ar/O_2_ DC | 0.25 | 2 | 1.15 | 19.99 | 0.78 | 17.91 | 1,666.67 | 5.59 |
| Ar/O_2_ DC | 0.25 | 3 | 1.09 | 19.22 | 0.74 | 15.38 | 1,666.67 | 7.41 |
| Ar/O_2_ DC | 0.25 | 4 | 1.07 | 19.22 | 0.71 | 14.53 | 1,666.67 | 10.56 |
| Average | 0.25 |  | 1.10 | 19.60 | 0.74 | 15.91 | 1,607.14 | 8.01 |
| Ar/O_2_ DC | 1.00 | 1 | 1.11 | 15.13 | 0.67 | 11.17 | 228.57 | 11.25 |
| Ar/O_2_ DC | 1.00 | 2 | 0.79 | 22.66 | 0.63 | 11.27 | 49.81 | 9.41 |
| Average | 1.00 |  | 0.95 | 18.90 | 0.65 | 11.22 | 139.19 | 10.33 |

**References**

1. Abbasi, S. *et al.* Proper annealing process for a cost effective and superhydrophobic ambient-atmosphere fabricated perovskite solar cell. *Mater. Sci. Semicond. Process.* **155**, 107241 (2023).

2. Amratisha, K. *et al.* Graded multilayer triple cation perovskites for high speed and detectivity self-powered photodetector via scalable spray coating process. *Sci. Rep.* **12**, 11058 (2022).
